# Therapeutic Potential of *Rosa davurica* Pall. Root Extract as an Antidiabetic Agent: A Comprehensive Analysis from Molecular Mechanisms to In Vivo Efficacy

**DOI:** 10.3390/ijms25168944

**Published:** 2024-08-16

**Authors:** Du Hyeon Hwang, Ravi Deva Asirvatham, Ramachandran Loganathan Mohan Prakash, Changkeun Kang, Euikyung Kim

**Affiliations:** 1 Department of Pharmacology and Toxicology, College of Veterinary Medicine, Gyeongsang National University, Jinju 52828, Republic of Korea; pooh9922@hanmail.net (D.H.H.); devabiochem@gnu.ac.kr (R.D.A.); mohanprakash111@gmail.com (R.L.M.P.); ckkang@gnu.ac.kr (C.K.); 2 Institute of Animal Medicine, Gyeongsang National University, Jinju 52828, Republic of Korea

**Keywords:** *Rosa davurica* Pall., STZ, PI3K, AMPK, antioxidant, diabetic rat

## Abstract

*Rosa davurica* Pall. is widely used in traditional oriental herbal therapy, but its components and molecular mechanisms of action remain unclear. This study investigates the antidiabetic potential of *Rosa davurica* Pall. root extract (RDR) and elucidates its underlying molecular mechanisms with in vitro and in vivo models. Data from the current study show that RDR exhibits strong antioxidant activity and glucose homeostasis regulatory effects. It significantly impacts glucose homeostasis in C2C12 skeletal muscle cells by inhibiting α-glucosidase activity. Further molecular mechanistic studies revealed that RDR promoted glucose uptake by phosphorylation of AMP-activated protein kinase (AMPK)/acetyl-CoA carboxylase (ACC), but not Phosphatidylinositol 3-kinase (PI 3-kinase)/Akt in C2C12 skeletal muscle cells. These actions increased the expression and translocation of glucose transporter type 4 (GLUT4) to the plasma membrane. In addition, RDR treatment in the STZ-induced diabetic rats remarkably improved the low body weight, polydipsia, polyphagia, hyperglycemia, and islet architecture and increased the insulin/glucose ratio. The liver (ALT and AST) and kidney marker enzyme (BUN and creatinine) levels were restored by RDR treatment as well. Phytochemical analysis identified eight major constituents in RDR, crucial for its antioxidant and antidiabetic activity. Through the molecular docking of representative glucose transporter GLUT4 with these compounds, it was confirmed that the components of RDR had a significantly high binding score in terms of structural binding. These findings from the current study highlight the antidiabetic effects of RDR. Collectively, our data suggest that RDR might be a potential pharmaceutical natural product for diabetic patients.

## 1. Introduction

Reactive oxygen species (ROS) generated during cellular aerobic metabolism have been implicated in both physiological and pathophysiological processes within the human body. Their close association with an array of chronic diseases, including rheumatism, cardiovascular disease, cancer, nephritis, ischemia, Parkinson’s neurodegenerative disease, and diabetes mellitus (DM), underscores their multifaceted impact [1,2]. DM, a pervasive chronic metabolic disorder characterized by hyperglycemia and inflammatory responses, affects over 170 million individuals globally [3]. A plethora of studies have underscored the substantial roles played by oxidative stress and inflammation in the pathogenesis of DM and its associated complications [4]. The intimate connection between abnormal glucose metabolism and DM manifests in impairments in insulin secretion and action. Skeletal muscles, constituting approximately 80% of insulin-mediated glucose uptake in the postprandial state, emerge as pivotal insulin target tissues crucial for maintaining glucose homeostasis [5,6,7,8]. The regulation of glucose transport pathways in skeletal muscle involves phosphatidylinositol-3 kinase (PI3K) and 5′-AMP-activated protein kinase (AMPK). Insulin signaling, which instigates increased glucose uptake in skeletal muscle, commences by activating PI3K and Akt (protein kinase B) [6]. This pathway orchestrates the activation of lipid kinase PI3K and the serine/threonine kinase Akt/PKB, leading to the translocation of glucose transporter GLUT4 from intracellular storage sites to the plasma membrane, thereby facilitating glucose entry into muscle cells [7,8,9]. Impairment of the PI3K/Akt signaling cascade results in diminished insulin-induced glucose transport into skeletal muscle cells [10,11]. Conversely, AMPK, a pivotal energy sensor, stimulates intracellular glucose uptake via an insulin-independent pathway [12]. Its activation leads to the phosphorylation of downstream sensors, including acetyl-CoA carboxylase (ACC) [13]. The pharmacological and hormonal activation of AMPK, coupled with interventions such as exercise/contraction and antidiabetic drugs like metformin, accentuates its regulatory role in glucose homeostasis [14,15]. Various polyphenols, including naringenin [16] and resveratrol [17], are recognized activators of AMPK. As the quest for therapeutic agents with high efficacy and minimal side effects intensifies, natural plant-based medicines are gaining prominence, offering limited or no adverse effects. The attention bestowed upon medicinal plants has surged due to their rich reservoirs of essential bioactive compounds, particularly flavonoids and phenolics, renowned for robust antioxidant and antidiabetic properties. In addition, these plant bioactive compounds are attracting attention as an antidiabetic treatment method because they act as α-glucosidase (α-d-glucoside glucohydrase) inhibitors, which delay glucose absorption and reduce postprandial plasma glucose levels. Consequently, a myriad of natural constituents are emerging as promising alternative treatments and potential therapeutic agents for diabetes [18,19]. *Rosa davurica* Pall. is a deciduous shrub classified taxonomically under the family Rosaceae. It is predominantly found in Korea, northeastern China, Siberia, Japan, and other regions of eastern Asia. The various parts of *R. davurica* Pall., including its leaves, stem, fruits, and roots, have been extensively utilized in traditional herbal medicine since ancient times. *R. davurica* is known to be rich in a wide range of beneficial components such as vitamins, flavonoids, tannins, and polyphenols [20,21]. The pharmacologically effective of *R. davurica* have already been documented, highlighting its antiviral, antibiotic, anti-HIV, anti-inflammatory, and antioxidant activities [22,23,24,25]. Despite the growing body of evidence supporting the multifaceted benefits of *R. davurica*, its potential antidiabetic effects and the underlying molecular mechanisms have remained elusive. This study endeavors to fill this gap by investigating the antidiabetic activity of *Rosa davurica* root extract (RDR) through a comprehensive analysis utilizing both in vitro and in vivo models, specifically C2C12 skeletal muscle cells and streptozotocin (STZ)-induced diabetic rats. Beyond exploring the pharmacological aspects, we delve into the intricate signaling cascades associated with RDR’s mechanism of action. Additionally, this study aims to identify the phytochemical components responsible for the observed effects, shedding light on the potential of RDR as an antidiabetic therapeutic agent.

## 2. Results

### 2.1. Polyphenol Content of RDR and Its Antioxidant and Radical Scavenging Activities

**Figure 1:**
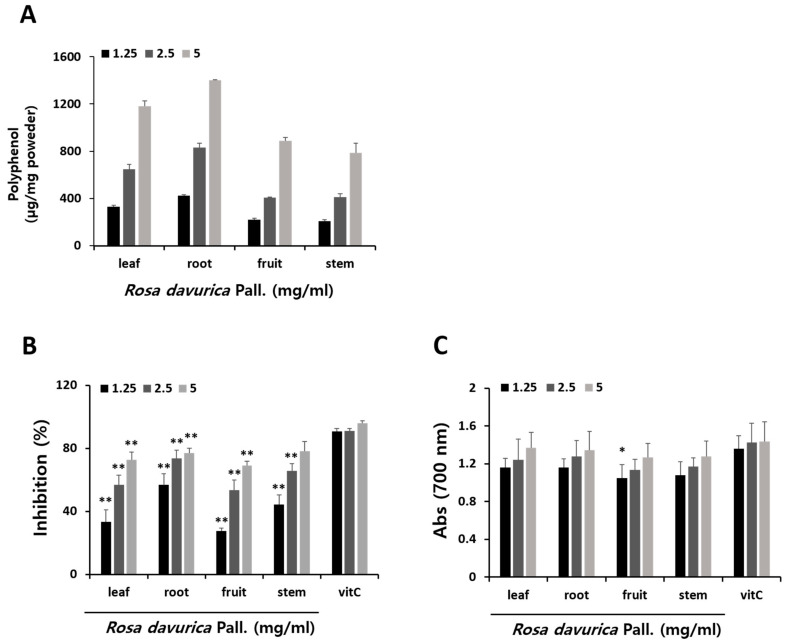
Comparisons of total polyphenol, antioxidant activities, and radical scavenging activities of methanol extraction in various parts of *Rosa davurica* Pall. with ascorbic acid. (**A**) Total polyphenol content of various parts of *Rosa davurica* Pall. (**B**) DPPH free radical scavenging assay. (**C**) Reducing power activity. The results are expressed as mean ± SD of three experiments. All experiments were performed in triplicate. Significant differences from the control group are indicated as * *p* < 0.05 and ** *p* < 0.01.

The polyphenol content of methanol extracts from various parts of *R. davurica* Pall. were determined, as illustrated in Figure 1A. Notably, the root extract of *R. davurica* Pall. (RDR) exhibited a markedly higher polyphenol content compared to other parts. To evaluate antioxidant effects, DPPH assay and reducing power activity assay were conducted on different segments of *R. davurica* Pall. (Figure 1B,C). The DPPH assay revealed a reduction in DPPH radicals attributed to the reactive oxygen species scavenging capability of *R. davurica* Pall., with comparisons made to ascorbic acid (used as a positive control). Furthermore, *R. davurica* Pall. demonstrated a robust reducing power. The polyphenol content values exhibited a positive correlation with both DPPH scavenging activity and reducing power. In alignment with these observations, RDR demonstrated the most potent radical scavenging effect in the DPPH assay and displayed significant reducing power activity, underscoring its effectiveness as a strong antioxidant.

### 2.2. α-Glucosidase Inhibitory Activities of RDR

**Figure 2:**
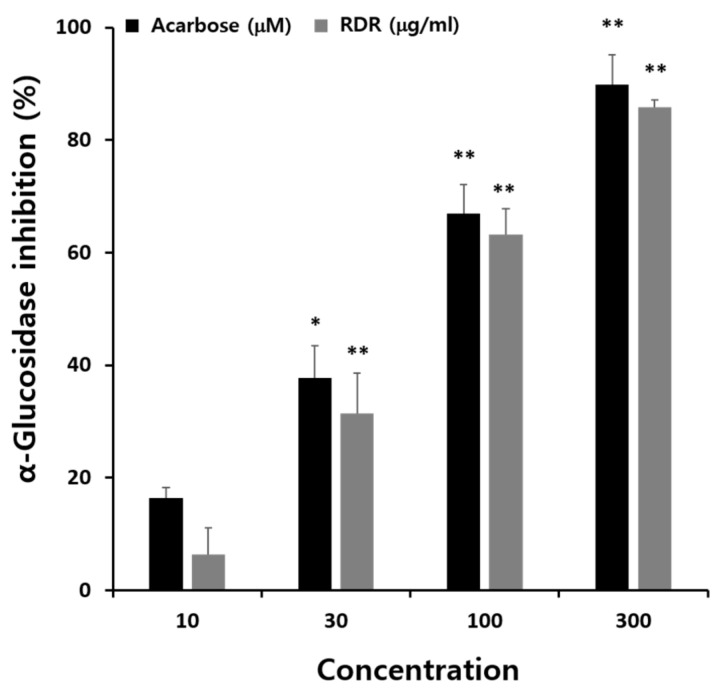
α-Glucosidase inhibitory activities of methanol extract in *R. davurica* Pall. root extract (RDR). The concentration of methanol extract in RDR (μg/mL) and acarbose (positive control; μM) are 10, 30, 100, and 300. The results are expressed as mean ± SD. All experiments were performed in triplicate. Significant differences from the control group are indicated as * *p* < 0.05 and ** *p* < 0.01.

The α-glucosidase inhibitory activities of RDR were evaluated using the methods described previously [26] (Figure 2). The findings demonstrated a dose-dependent inhibition of α-glucosidase activity by RDR. The maximum inhibitory effect of RDR was noted to be 85.9% at a concentration of 300 μg/mL, comparable to the maximum inhibitory activity observed with the positive control, acarbose. Acarbose exhibited an inhibitory effect of 89.87% at 300 μM.

### 2.3. Effects of RDR on Glucose Uptake in C2C12 Cells

**Figure 3:**
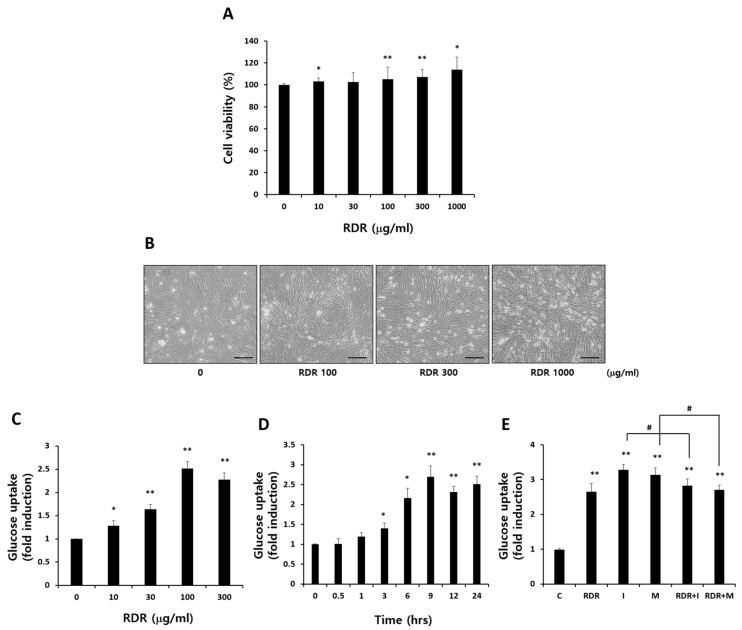
Effects of RDR on glucose uptake in C2C12 cells. (**A**) Effect of various concentrations of RDR on the viability in C2C12 cells. Exponentially growing C2C12 cells were plated onto a 24-well plate and treated with indicated various concentrations (0–1000 μg/mL) of RDR for 24 h. The viability was assessed by MTT assay. (**B**) Effect of RDR on C2C12 cell morphology. Scale bars represent 100 μm. (**C**) Effects of RDR on glucose uptake. The cells were incubated without or with 10, 30, 100, and 300 μg/mL RDR for 24 h. (**D**) Effects of RDR on glucose uptake (time-course). (**E**) C2C12 cells were incubated without (control, C) or with 100 μg/mL RDR (9 h), 100 nM insulin (I) (0.5 h), and 2 mM metformin (M) (2 h) followed by glucose uptake measurements. The results are expressed as mean ± SD. All experiments were performed in triplicate. Significant differences from the control group are indicated as * *p* < 0.05, ** *p* < 0.01, and # *p* < 0.05 vs. insulin or metformin alone.

To evaluate the potential cytotoxic effects of RDR, both cell morphology and viability were examined initially. Cell viability was assessed using the MTT assay, with C2C12 cells treated with various RDR concentrations (ranging from 0 to 1000 µg/mL) for 24 h. As depicted in Figure 3A,B, RDR did not induce any toxic effects on cell morphology or viability across all concentrations tested. Consequently, concentrations of 10 to 300 μg/mL of RDR were chosen for a 24 h treatment period, as these concentrations exhibited no adverse effects on the cells.

To explore the impact of RDR on glucose uptake in skeletal muscle cells, C2C12 cells were treated with varying RDR concentrations for 24 h. As shown in Figure 3C, RDR demonstrated a dose-dependent increase in glucose uptake. Significant enhancement occurred at 10 μg/mL RDR (1.28-fold of control, *p* < 0.05), with maximum stimulation observed at 100 μg/mL RDR (2.52-fold of control, *p* < 0.01). However, RDR-treated groups (300 μg/kg) showed a slight reduction in glucose uptake.

Further, a time-course analysis was conducted by incubating C2C12 cells with 100 μg/mL of RDR for durations ranging from 30 min to 24 h (Figure 3D). The maximum stimulation in glucose uptake was achieved after 9 h of RDR exposure (2.7-fold of control, *p* < 0.01). In a comparative analysis with widely used therapeutic agents for diabetes mellitus, metformin and insulin (Figure 3E), RDR-induced glucose uptake (2.66-fold of control, *p* < 0.01) was found to be comparable to the increases observed with insulin (3.28-fold of control, *p* < 0.01) and metformin (3.14-fold of control, *p* < 0.01) treatments. Notably, RDR did not exert a synergistic effect on insulin and metformin-stimulated glucose uptake. These findings suggest that RDR has the potential to enhance muscle cell glucose uptake, possibly acting through signaling pathways associated with glucose uptake in C2C12 cells.

### 2.4. Effects of RDR on the PI3K/Akt Signaling Cascade

**Figure 4:**
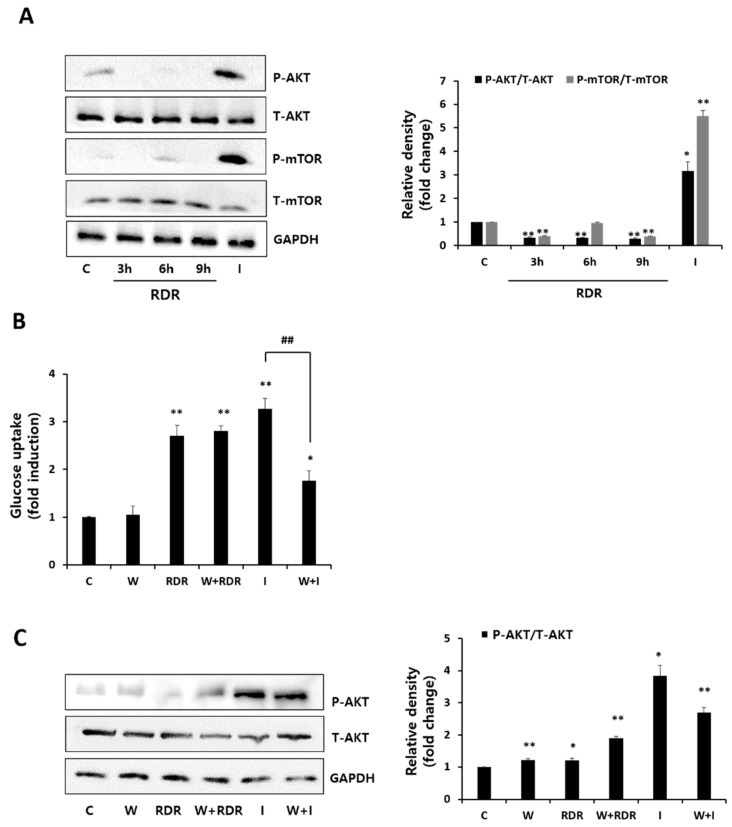
Effects of RDR on the PI3K/Akt signaling cascade. (**A**) Effects of RDR on the Akt signaling cascade. The cells were incubated without (control, C) or with 100 μg/mL RDR (3, 6, and 9 h) or with 100 nM insulin (I) (15 min). The cell lysates were western blotted for total Akt, phospho-Akt, total mTOR, phospho-mTOR, or GAPDH. (**B**) Effect of wortmannin on RDR-induced glucose uptake. (**C**) Effects of RDR on the Akt signaling cascade by Western blot analysis. The cells were incubated in the absence (control, C) or presence of 100 nM wortmannin (W) for 15 min, followed by treatment with or without 100 μg/mL RDR for 9 h, or 100 nM insulin (I) for 0.5 h. The results are expressed as mean ± SD. All experiments were performed in triplicate. Significant differences from the control group are indicated as * *p* < 0.05, ** *p* < 0.01, and ## *p* < 0.01 vs. insulin alone.

To unravel the mechanism behind the increased glucose uptake induced by RDR in skeletal muscle cells, we examined the phosphorylation levels of the PI3K/Akt signaling pathway, a pivotal player in insulin-stimulated glucose uptake. Insulin activates PI3K, leading to downstream Akt activation, crucial for facilitating glucose uptake in response to insulin. We investigated the effect of RDR on PI3K/Akt expression through Western blot analysis (Figure 4A). Contrary to the significant Akt expression observed with insulin treatment, RDR did not affect Akt expression. Moreover, RDR treatment did not influence mTOR phosphorylation (a downstream indicator of Akt activation), while insulin treatment resulted in a noteworthy increase in mTOR phosphorylation (Figure 4A). To further validate the involvement of PI3K in RDR’s action, we employed the PI3K inhibitor wortmannin. As depicted in Figure 4B, wortmannin effectively blocked insulin-stimulated glucose uptake (I: 3.27-fold of control, W + I: 1.77-fold of control). However, wortmannin had no impact on RDR-stimulated glucose uptake (RDR: 2.71-fold of control, W + RDR: 2.81-fold of control), indicating that PI3K signaling is not implicated in RDR’s action. Additionally, we assessed the effect of RDR on Akt expression. As expected, RDR did not influence Akt expression, in contrast to the significant Akt expression observed with insulin stimulation in C2C12 cells (Figure 4C). In summary, our data suggest that RDR has no effect on the PI3K/Akt signaling pathway.

### 2.5. Effects of RDR on AMPK Signaling

**Figure 5:**
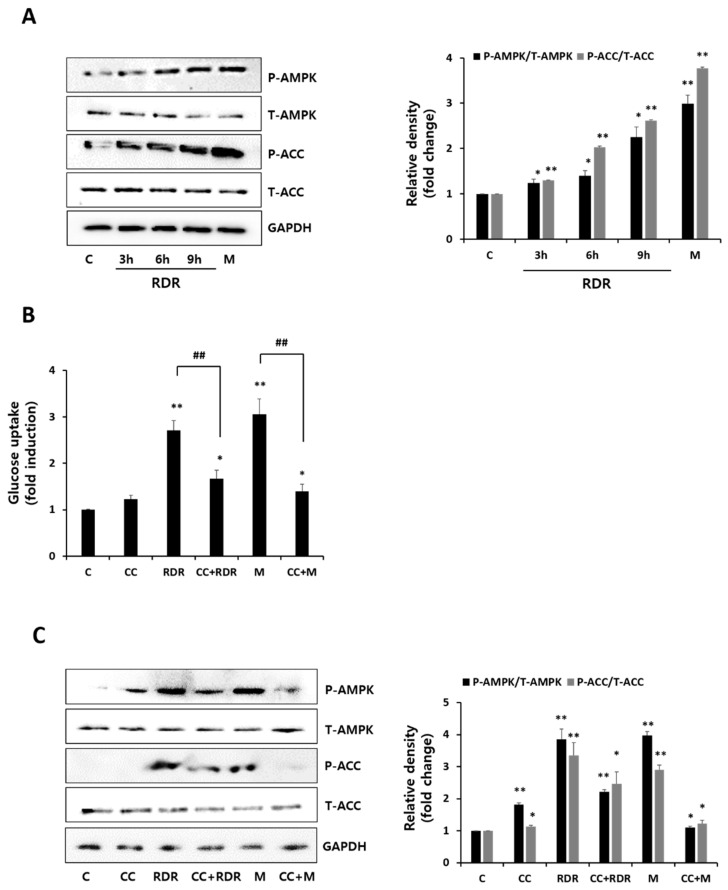
Effects of RDR on the AMP-activated protein kinase (AMPK) signaling cascade. (**A**) The cell lysates from cells treated without (control, C) or with 100 μg/mL RDR (3, 6, and 9 h) or with 2 mM metformin (M) (2 h) were prepared and Western blotted for phospho-AMPK, total AMPK, phospho-ACC, total ACC, or GAPDH. (**B**) Effect of the AMPK inhibitor compound C (CC) on RDR-induced glucose uptake. (**C**) Effects of RDR on the AMPK signaling cascade by Western blot analysis. Cells were incubated in the absence (control, C) or presence of 25 μM compound C (CC) for 0.5 h, followed by exposure to 100 μg/mL RDR for 9 h or 2 mM metformin (M) (2 h). The results are expressed as mean ± SD. All experiments were performed in triplicate. Significant differences from the control group are indicated as * *p* < 0.05, ** *p* < 0.01, and ## *p* < 0.01 vs. RDR or metformin alone.

To evaluate the impact of RDR on AMPK phosphorylation, we conducted experiments to examine the levels of AMPK activation (Figure 5A). Incubation with RDR in a time-dependent manner resulted in a noticeable increase in AMPK activation. The phosphorylation of acetyl-CoA carboxylase (ACC), a downstream physiological target of AMPK serving as an indicator of AMPK activation, was significantly enhanced by both RDR and metformin. Subsequently, we explored the effect of the AMPK inhibitor compound C (CC) on RDR-induced glucose uptake (Figure 5B). Remarkably, CC prominently inhibited RDR-induced glucose uptake (RDR: 2.71-fold of control, CC + RDR: 1.67-fold of control), and the metformin-induced glucose uptake was similarly significantly inhibited (M: 3.1-fold of control, CC + M: 1.4-fold of control). We further investigated the influence of CC on the RDR-induced phosphorylation of AMPK and ACC (Figure 5C). Consequently, CC impeded the RDR-induced phosphorylation of AMPK and ACC. These findings suggest that RDR may induce glucose uptake in skeletal muscle cells through mechanisms involving the AMPK/ACC signaling pathways.

### 2.6. Effects of RDR on Glucose Transporter Type 4

**Figure 6:**
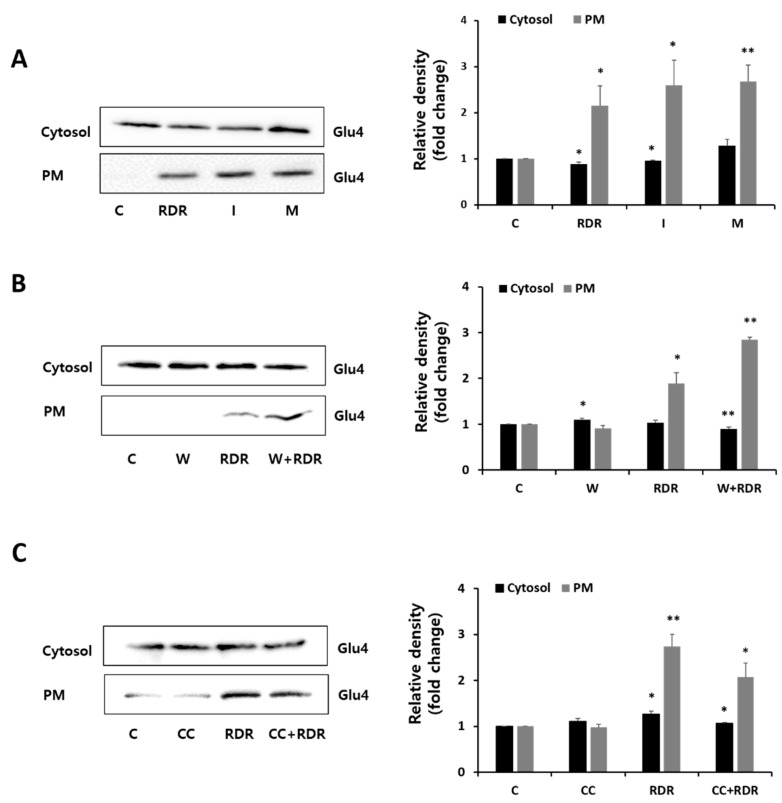
Effects of RDR on glucose transporter type 4 (GLUT4). (**A**–**C**) C2C12 cells were incubated without (control, C) or with 100 μg/mL RDR (9 h), with 100 nM insulin (I) (0.5 h), with 2 mM metformin (M) (2 h), with 25 μM compound C (CC) (0.5 h), or with 100 nM wortmannin (W) (15 min). Protein extracts of cytosol and plasma membrane (PM) fractions were prepared and subjected to Western blot assay using the primary antibodies GLUT4. The results are expressed as mean ± SD. All experiments were performed in triplicate. Significant differences from the control group are indicated as * *p* < 0.05 and ** *p* < 0.01.

In skeletal muscle, glucose transporter type 4 (GLUT4) plays a crucial role as the primary transporter within the GLUT family, facilitating glucose uptake through the activation of Akt and AMPK [27,28]. To delve deeper into the mechanism by which RDR increased glucose uptake, we conducted an analysis of GLUT4 expression in the cytosol and plasma membrane (PM) fractions of C2C12 cells (Figure 6). Remarkably, treatment with RDR significantly elevated PM–GLUT4 protein levels, similar to insulin and metformin (Figure 6A). Importantly, the translocation of GLUT4 glucose transporters mediated by RDR was not impacted by the presence of the PI3K inhibitor wortmannin, suggesting the irrelevance of the PI3K/Akt cascade in the action of RDR (Figure 6B). Conversely, the presence of the AMPK inhibitor CC substantially reduced the RDR-induced translocation of GLUT4 glucose transporters (Figure 6C). Collectively, these findings indicate that RDR enhances GLUT4 expression in C2C12 cells by activating the AMPK pathway.

### 2.7. Effect of RDR on Symptoms of Diabetes Mellitus (DM) in STZ-Induced Diabetic Rats

**Figure 7:**
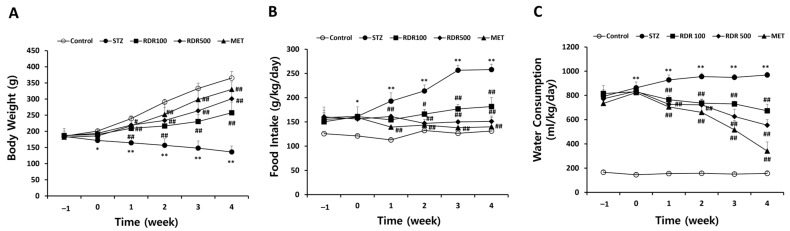
Effects of RDR on symptoms of diabetes mellitus (DM) in STZ-induced diabetic rats. (**A**) Changes in body weight, (**B**) food intake, and (**C**) water consumption every week. Throughout the experiments, all rats were monitored and recorded daily and/or weekly for the symptoms. The results are expressed as mean ± SD. Significant differences from the control group are indicated as * *p* < 0.05 or ** *p* < 0.01, and differences from the STZ group are indicated as # *p* < 0.05 or ## *p* < 0.01.

To assess the therapeutic impact of RDR on diabetes in vivo, we employed streptozotocin (STZ)-induced diabetic rat models. Throughout the experimental period, the control group exhibited a continuous increase in weight, food intake, and water consumption, whereas the STZ-induced group experienced significant decreases in these parameters (Figure 7). At the conclusion of the study, the RDR-treated groups (at doses of 100 and 500 mg/kg) demonstrated a noteworthy increase in weight by approximately 121.2 ± 33.34 g and 164 ± 29.99 g, respectively, compared to the STZ-induced group. Notably, the MET-treated group exhibited a significantly higher weight (194 ± 22.98 g) compared to the STZ-induced group (Figure 7A). In addition, the RDR-treated groups (100 and 500 mg/kg) showed a substantial reduction in both food intake and water consumption, reaching levels of (29.56% and 41.44%) and (30.6% and 42.67%), respectively. Similarly, the MET-treated group displayed effective reductions compared to the STZ-induced group, with decreases of (45.75% and 64.78%) in food intake and water consumption, respectively (Figure 7B,C).

### 2.8. Effects of RDR on Blood Glucose and Liver and Kidney Functions in STZ-Induced Diabetic Rats

**Figure 8:**
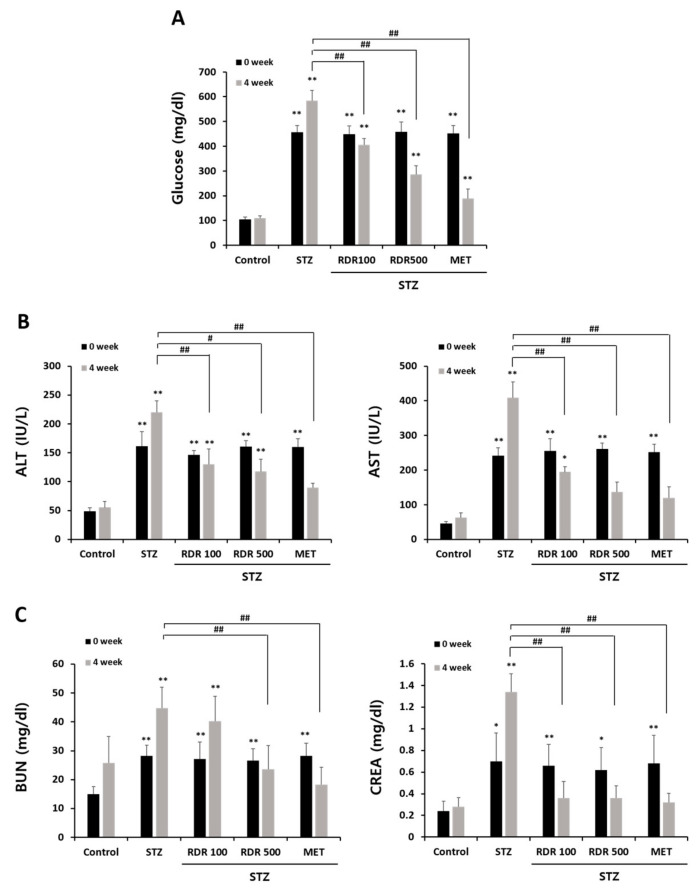
Effects of RDR on blood glucose and liver and kidney functions in STZ-induced diabetic rats. (**A**) The levels of blood glucose. (**B**) The level of liver enzyme markers (alanine transaminase (ALT) and aspartate transaminase (AST)). (**C**) The level of kidney enzyme markers (BUN and serum creatinine). The results are expressed as mean ± SD. Significant differences from the control group are indicated as * *p* < 0.05 or ** *p* < 0.01, and differences from the STZ group are indicated as # *p* < 0.05 or ## *p* < 0.01.

Throughout the experiment, the glucose levels in the STZ group exhibited a continuous increase. As illustrated in Figure 8A, upon completion of the 4-week treatment period, the glucose levels in the STZ group were 5.4-fold higher than those in the control group. However, treatment with RDR (100 and 500 mg/kg) or MET resulted in a significant reduction in glucose levels (29.8%, 52.1%, and 73%, respectively). These findings underscore the potential of RDR to ameliorate elevated blood glucose levels in STZ-induced diabetic rats. In the context of diabetes mellitus (DM), elevated blood glucose levels can lead to damage to vital organs such as the liver and kidneys. Therefore, we investigated the impact of RDR on liver and kidney functions in STZ-induced diabetic rats. The results presented in Figure 8A depict the analysis of liver and kidney enzymes in STZ-induced diabetic rats. Levels of liver enzymes (ALT and AST) and kidney marker enzymes (BUN and creatinine) were significantly elevated in the STZ group (Figure 8B,C). Conversely, treatment with RDR (100 and 500 mg/kg) or MET exhibited a substantial restoration in ALT levels (130.0 ± 26.45, 117.8 ± 21.08, and 89.4 ± 8.08 IU/L, respectively). This trend was consistently observed in the levels of AST, BUN, and creatinine. Therefore, RDR may offer protective effects against diabetes and its associated complications.

### 2.9. Effects of RDR on Pancreatic Function in STZ-Induced Diabetic Rats

**Figure 9:**
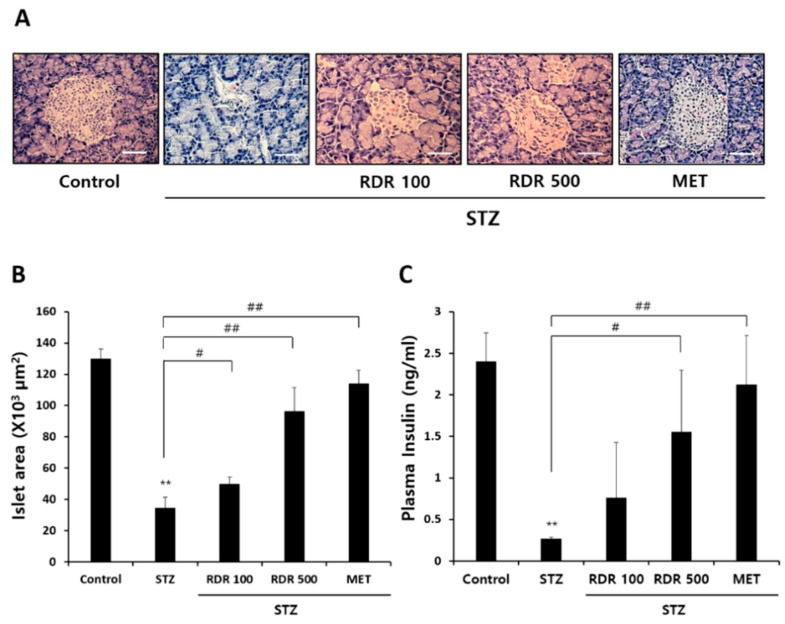
Effects of RDR on plasma insulin and histopathological changes of pancreatic tissue in STZ-induced diabetic rats. (**A**) Histopathological changes of pancreatic tissue stained with hematoxylin and eosin (H&E) stain (magnification, 200×; scale bars, 100 μm). (**B**) Analysis of the islet area in the pancreas stained with H&E stain. The islet area was determined from at least 5 different islets per pancreas stained. (**C**) Plasma insulin was determined using a rat insulin ELISA kit. The results are expressed as mean ± SD. Significant differences from the control group are indicated as ** *p* < 0.01, and differences from the STZ group are indicated as # *p* < 0.05 or ## *p* < 0.01.

The pancreatic islets were particularly investigated to elucidate the role of RDR in ameliorating STZ-induced diabetes and its therapeutic impact on insulin secretion. Histopathological changes in the pancreatic islets of STZ-induced diabetic rats were assessed to evaluate the efficacy of RDR treatment. As depicted in Figure 9A, microscopic examination of the control group revealed normal pancreatic tissues with insulin-producing β-cells centrally located. In contrast, the STZ-treated group exhibited partial degeneration and shrinkage in the islets of Langerhans. Conversely, RDR treatment groups showed partial reversal (though not complete) of the STZ-induced reduction in islet size (Figure 9B). Furthermore, we measured the level of plasma insulin in the blood (Figure 9C). Consistent with the histopathological changes, the STZ-treated group displayed a significant decrease in plasma insulin compared to the control group. The RDR (100 and 500 mg/kg)-treated groups demonstrated a substantial recovery in plasma insulin levels (0.76 ± 0.67 and 1.56 ± 0.74 mg/mL, respectively). The MET-treated group brought insulin levels close to those of the control group. These collective findings strongly support the potential of RDR as a promising candidate for the treatment of diabetes mellitus.

### 2.10. Identification of Phytochemical Compounds in RDR through GC–MS Chromatogram

**Figure 10:**
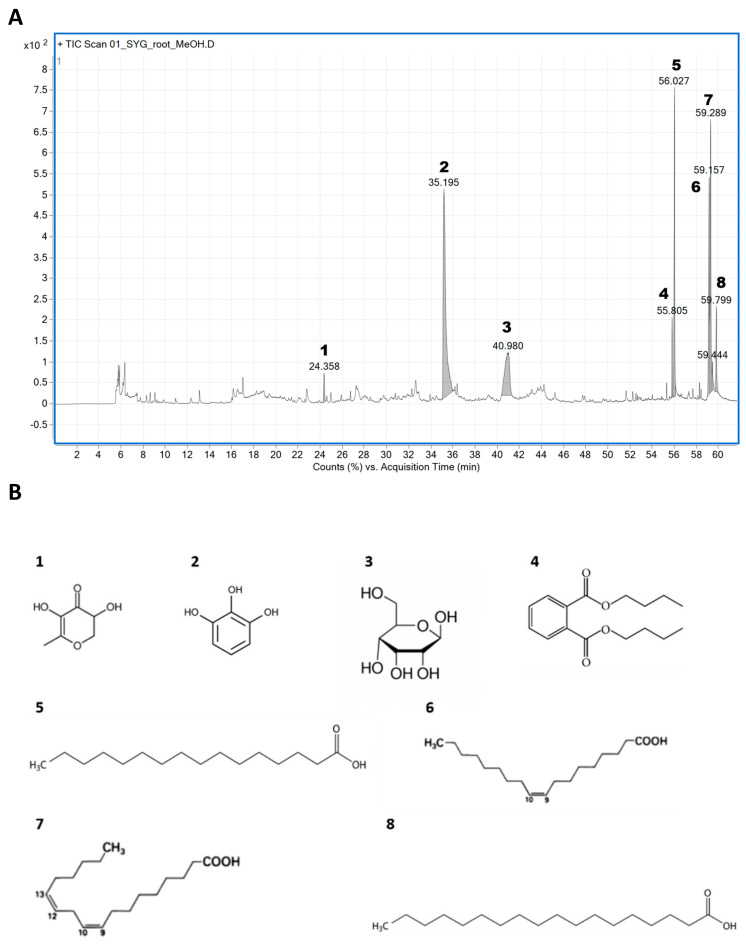
(**A**) GC–MS chromatogram of the methanol extract of RDR. (**B**) The main compounds identified in RDR: **1**. 4H-pyran-4-one, 2,3-dihydro-3,5-dihydroxy-6-methyl-, **2**. 1,2,3-benzenetriol, **3**. D-allose, **4**. dibutyl phthalate, **5**. n-hexadecanoic acid, **6**. 9,12-octadecadienoic acid (Z,Z)-, **7**. oleic acid, and **8**. octadecanoic acid.

In order to pinpoint the specific compounds in RDR accountable for its potential antidiabetic effects, we employed GC–MS analysis to determine the phytochemical constituents present in the RDR extract (Figure 10). The analysis unveiled the presence of eight distinct phytochemical constituents in RDR, summarized in Table 1, along with their corresponding retention time (Rt) and reported activities. The prominent constituents identified in the extract included 4H-pyran-4-one, 2,3-dihydro-3,5-dihydroxy-6-methyl-(**1**), 1,2,3-benzenetriol (**2**), D-allose (**3**), dibutyl phthalate (**4**), n-hexadecanoic acid (**5**), 9,12-octadecadienoic acid (Z,Z)-(**6**), oleic acid (**7**), and octadecadienoic acid (**8**). Notably, these constituents exhibited a high degree of similarity, with over 90% matching the MS library in the NIST software (https://webbook.nist.gov/chemistry/cas-ser/, accessed on 26 June 2024). Additionally, these eight main compounds have been previously documented in Dr. Duke’s database for their antioxidant, antimicrobial, and antidiabetic properties and detailed analysis conditions were shown in Supplementary Table S1.

### 2.11. Molecular Docking Analysis of the Potential Interaction between the Main Components in RDR and GLUT4

**Figure 11:**
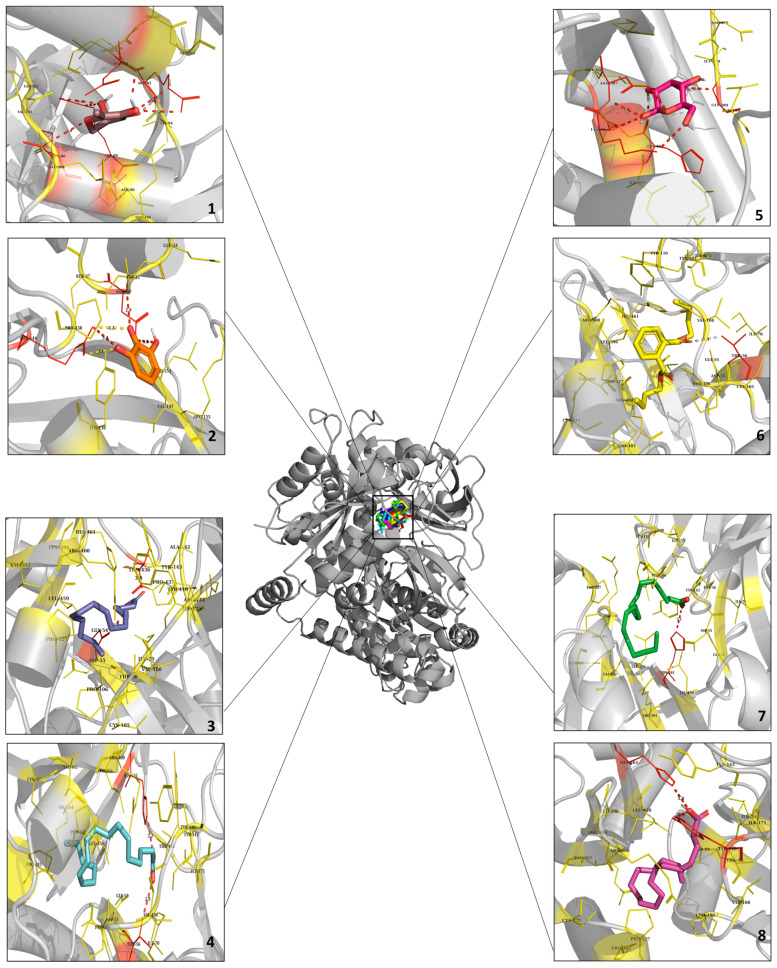
Molecular docking analysis of GLUT4 and the main chemical compounds in RDR. Prediction of ligand binding sites on GLUT4 and cartoon representation of binding pocket residues visualized using AutoDock Vina and PyMOL. The structure of GLUT4 was bound efficiently to **1**. 4H-pyran-4-one, 2,3-dihydro-3,5-dihydroxy-6-methyl- (Tint), **2**. 1,2,3-benzenetriol (Orange), **3**. D-allose (Red), **4**. dibutyl phthalate (Yellow), **5**. n-hexadecanoic acid (Blue), **6**. 9,12-octadecadienoic acid (Z,Z)-(Cyan), **7**. oleic acid (Green), and **8**. octadecanoic acid (Megenta).

The eight primary compounds identified in RDR underwent molecular docking analysis to investigate their binding modes at the GLUT4 receptor. As detailed in Table 2, nearly all of these compounds demonstrated a favorable binding affinity for GLUT4. Docking image is presented in Figure 11, Additionally, Supplementary Figure S1 exhibits the 2D docking image obtained from the Discovery Studio visualizer, and pymol was used to differentiate each ligand color-coded for clarity. With detailed hydrogen bonds and hydrophobic interaction. The chemical compounds were systematically evaluated for their binding affinities and interactions with specific amino acid residues within the GLUT4 receptor. Notably, 4H-pyran-4-one, 2,3-dihydro-3,5-dihydroxy-6-methyl, or DDMP (**1**), exhibited a binding affinity of −5.5 kcal/mol, interacting with HIS-384, LYS-386, ASN-387, and ILE-479. Pyrogallol, also recognized as 1,2,3-benzenetriol (**2**), displayed a binding affinity of −5.6 kcal/mol, forming interactions with ASN-36 and ARG-144. Conversely, D-allose (**3**) demonstrated the highest binding affinity of −6.9 kcal/mol, establishing interactions with THR-56, TYR-110, and ARG-400, among others. n-Hexadecanoic acid, or palmitic acid (**4**), exhibited a binding affinity of −6.3 kcal/mol, interacting with GLY-54, THR-436, and additional residues including ILE-70, TYR-110, ILE-175, PRO-327, LEU-396, VAL-402, and LEU-459. Dibutyl phthalate (**5**) displayed interactions particularly with THR-56, and additional interactions were with TYR-110, TYR-143, ILE-175, PRO-327, LEU-396, ARG-400, VAL-402, and PRO-483. 9,12-Octadecadienoic acid (Z,Z)-, also recognized as linoleic acid (**6**), exhibited a binding affinity of −6.0 kcal/mol and interacted with THR-56, PRO-106, TYR-110, ILE-175, PRO-327, LEU-396, VAL-402, LEU-459, and PRO-483. Oleic acid (**7**) interacted with TYR-110, PRO-327, LEU-396, ARG-400, VAL-402, LEU-459, and PRO-483, displaying a binding affinity of −5.7 kcal/mol. Last but not least, octadecanoic acid, also referred to as stearic acid (**8**), primarily interacted with TYR-110 and additionally with ILE-175, PRO-327, and LEU-396. These findings highlight varying levels of specific amino acid connections and affinities that these chemicals exhibit with proteins, potentially influencing the biological or chemical functions of proteins. The docking results strongly suggest a robust interaction between RDR, containing a diverse array of phytochemical compounds, and GLUT4. Consequently, we propose that RDR may prove to be an effective antidiabetic drug based on predicted docking data, complemented by in vitro and in vivo results.

## 3. Discussion

As the global prevalence of diabetes continues to rise, public interest in this disease has grown significantly. Over recent decades, diabetes mellitus (DM) has become a formidable threat to human health, posing a substantial burden on public health globally [2]. While recent antidiabetic therapeutic strategies have demonstrated efficacy, challenges related to side effects and tolerability persist. Therefore, the development of safer and more effective antidiabetic medications with limited adverse effects is imperative. Researchers are increasingly drawn to natural products and their bioactive compounds for novel DM treatments, given their favorable attributes such as low toxicity, efficacy, and availability. Natural compounds, plants, and their bioactive constituents have emerged as promising alternatives for therapeutic applications [29]. The plant species *R. davurica* Pall., investigated in this research, is renowned for its diverse biological effects. Among various part extracts studied, the root extract of *R. davurica* Pall. (RDR) exhibited significantly higher polyphenol content. Moreover, RDR demonstrated robust antioxidant activity in multiple in vitro assays, including assessments of polyphenols, DPPH scavenging, and reducing power, surpassing that of ascorbic acid (Figure 1). These observations directed our focus towards the polyphenols and antioxidant compounds present in RDR, given their potential health benefits attributed to antioxidant properties. In the current investigation, GC–MS analysis identified a total of eight phytochemical constituents in RDR. GC–MS analysis is a commonly used analytical technique for identifying components present in plant extracts. The technique can detect components in trace amounts, which is critical for understanding potential biological activities [30]. In conclusion, RDR encompasses a diverse range of bioactive constituents, exhibiting potent anti-inflammatory and antidiabetic activities and hypocholesterolemic effects (Table 1 and Figure 10).

Recent studies have highlighted the antidiabetic effects of polyphenols such as carnosic acid [31], rosmarinic acid [32], and berberine [33], a plant antioxidant commonly used in traditional oriental medicine. These compounds exert their effects by activating AMPK. Considering these findings collectively, the effective antioxidant and antidiabetic effects of RDR may indeed involve AMPK activation. Furthermore, polyphenols have been suggested to regulate hyperglycemia through various mechanisms, including inhibiting α-glucosidase activity in peripheral tissues to reduce glucose absorption [34,35,36], inducing adrenergic activation of muscle glucose uptake [37], and suppressing hepatic gluconeogenesis [38]. Taken together, it is plausible to suggest that the effective antioxidant and hypoglycemic effects of RDR may be attributed to its metabolic activity in skeletal muscle. The α-glucosidase enzyme plays a crucial role in carbohydrate digestion, breaking down disaccharides into simple sugars and leading to an increase in blood glucose levels. Thus, inhibitors of α-glucosidase, which catalyze cleavage of glucose from disaccharides, are effective in delaying glucose absorption and managing diabetes. Acarbose is widely used in the treatment of patients with diabetes via inhibiting, which converts complex polysaccharides into monosaccharides. However, gastrointestinal side effects, mainly abdominal discomfort and flatulence, have often been reported [39]. The inhibitory effects against α-glucosidase of the polyphenols-rich RDR were thus demonstrated in C2C12 cells, with acarbose serving as a positive control. The α-glucosidase maximum inhibitory effect of RDR was noted to be 85.9%, comparable to the maximum inhibitory activity observed with the positive control acarbose (Figure 2). Based on these results, RDR, apart from directly inhibiting α-glucosidase, might be effectively exploited in the management of hyperglycemia with minimal side effects. A significant finding of our investigation is that RDR promotes glucose uptake in skeletal muscle cells. The data revealed that RDR enhances glucose uptake in a dose-dependent manner (Figure 3C) and exhibits time-dependent effects (Figure 3D) in C2C12 skeletal muscle cells, indicating its potential role in regulating glucose metabolism within these cells. Notably, the extent of stimulation observed with RDR treatment approached the maximal effects elicited by insulin and metformin (Figure 3E). These findings suggest that, when insulin response is impaired, as in the case of insulin resistance, treatment with RDR may offer the potential to restore glucose uptake.

To gain insight into the signal pathways underlying RDR-induced glucose uptake, we conducted a Western blot analysis. RDR-induced glucose uptake was not affected by wortmannin, a PI3K irreversible inhibitor, indicating that PI3K is not involved in the mechanism of action of RDR. Moreover, RDR did not affect the phosphorylation of mTOR [9], the downstream target of Akt, in contrast to a significant increase seen with insulin. While insulin-induced glucose uptake was significantly reduced by wortmannin, effectively blocking PI3K activation, our results clearly indicate that insulin-stimulated glucose uptake is dependent on the PI3K/Akt pathway, whereas RDR-induced glucose uptake is independent of this pathway (Figure 4). Another important signaling pathway involved in glucose uptake is AMPK, which phosphorylates downstream target proteins associated with fatty acid oxidation, lipid metabolism, and glucose uptake [40]. Remarkably, treatment with RDR significantly increased the phosphorylation of AMPK. Furthermore, RDR treatment also increased the phosphorylation of ACC, a downstream effector of AMPK frequently utilized as a surrogate marker for AMPK activity in various studies [41] (Figure 5A). To further investigate the involvement of AMPK in the action of RDR, we employed compound C, a specific AMPK inhibitor. Significantly, compound C markedly reduced RDR-induced glucose uptake, indicating the involvement of AMPK in RDR’s action. Moreover, compound C abolished the RDR-induced phosphorylation of AMPK and ACC, effectively inhibiting AMPK activity (Figure 5B,C). Furthermore, our findings suggest that RDR impacts glucose metabolism by regulating the plasma membrane receptor GLUT4, as demonstrated in Figure 6, where RDR treatment was observed to influence the translocation of GLUT4 to the plasma membrane. Overall, these results shed light on the potential signaling pathways involved in RDR-induced glucose uptake, highlighting the involvement of the AMPK pathway, as well as the regulation of GLUT4 in skeletal muscle cells.

Streptozotocin (STZ) is an antibiotic synthesized by *Streptomyces achromogenes* and is widely used to induce DM in experimental animal models. It has been demonstrated for its selective pancreatic β-cell cytotoxicity and associated with the generation of ROS, which causes oxidative damage, resulting in impaired glucose tolerance and eventually leading to hyperglycemia [42,43]. Numerous studies have shown that potential antioxidant compounds can protect pancreatic islets against the cytotoxic effects of STZ-inducing diabetes in animal models [44,45,46]. In the present study, we have demonstrated that RDR can improve diabetic features such as polydipsia, polyphagia, and reduced body weight (Figure 7), lower plasma glucose levels, and raise plasma insulin concentrations in STZ-induced diabetic rats (Figure 8A and Figure 9A). Interestingly, RDR clearly restored liver and kidney enzymes to near-normal levels (Figure 8B,C). The elevated enzyme levels were related to tissue damage, causing accumulation. RDR’s ability to repair liver and kidney enzymes, possibly related to its phenolic compounds and antioxidant activity, can play a crucial role in protecting against DM and chronic complications such as chronic kidney disease and cardiovascular diseases, which are associated with oxidative stress [18,19]. RDR treatment also protected the Langerhans (Figure 9B). The immunohistochemical results show that pancreatic β-cells are destroyed by STZ, whereas the RDR treatment group partially prevented degeneration and increased the area of insulin-immunoreactive β-cells. Taken together, the main findings of the current study suggest that the antioxidant potential of RDR leads to the possibility of antidiabetic activity. Therefore, RDR might be a potential pharmaceutical natural product for diabetic patients.

## 4. Materials and Methods

### 4.1. Chemicals and Reagents

Dulbecco’s Modified Eagle’s Medium (DMEM), Bovine serum albumin (BSA), Fetal bovine serum (FBS), penicillin–streptomycin–amphotericin B, and trypsin were obtained from Gibco-BRL (Grand Island, NY, USA). Dimethyl sulphoxide (DMSO), 2,2-diphenyl-1-picryl-hydrazyl (DPPH), Folin–Ciocalteu’s phenol reagent, gallic acid, L-ascorbic acid, 3-(4,5-dimethylthiazol-2-yl)-2,5-diphenyltetrazolium bromide (MTT), streptozotocin (STZ), metformin (MET), and yeast α-glucosidase were purchased from Sigma-Aldrich Inc. (St. Louis, MO, USA). Several antibodies were used in our study, including phospho- and total-PI3K, Akt, mTOR, AMPK, ACC, and GAPDH; they were from Cell Signaling Technology (Beverly, MA, USA). Glucose transporter type 4 (GLUT4) antibody was from Santa Cruz (Santa Cruz, CA, USA). Metformin was from Sigma (Oakville, ON, Canada). Insulin (Humulin R) was from Eli Lilly (Indianapolis, IN, USA). Compound C and wortmannin were purchased from Calbiochem (Gibbstown, NJ, USA). All reagents used were of the purest grade available.

### 4.2. Preparation of R. davurica Pall. Extracts

The specimens of *R. davurica* Pall. were obtained from Jungsun, Gangwon-do, Republic of Korea in November 2020. The identity of the plant was confirmed by Dr. E. Kim, professor in the Department of Pharmacology and Toxicology, Gyeongsang National University, Jinju, Republic of Korea. The samples were cut into leaf, stem, root, and fruit parts. Each different part of the samples was washed and dried at room temperature. The dried samples were then ground into fine powders. An aliquot (10 g) of the powder samples was added to 200 mL of 70% methanol at room temperature, and shaken (150 rpm) for 24 h. After the extraction, each extract was filtered through the Advantech No. 3 filter paper (Osaka, Japan). The filtered liquid was evaporated using a rotary vacuum evaporator (Tokyo Rikakikai Co., Ltd., Tokyo, Japan). The final product was lyophilized and kept at −70 °C until use.

### 4.3. Determination of Total Polyphenols

Total polyphenol contents were assessed as described in [47]. In brief, for the polyphenol standard, gallic acid (1 mg/mL) was diluted to the indicated concentrations of each standard (0, 3.125, 6.25, 12.5, 25, 50, 100, and 200 μg/mL), respectively. Various parts of *R. davurica* Pall. in powder (sample) forms were freshly dissolved in 1 mL of distilled water. To assess the total polyphenol contents, 10 μL aliquots of each sample and standard solution were mixed with 50 μL of Folin–Ciocalteu reagent and incubated for 5 min. Then, they were added with 40 μL of sodium carbonate solution (7.5%) and incubated for an additional 2 h in the dark room. Their absorbances were measured at 750 nm using a GENios^®^ spectrophotometer (PowerWave^TM^XS, BioTek Instruments, Inc., Winooski, VT, USA).

### 4.4. DPPH Radical Scavenging Assay

The free radical scavenging abilities of various parts of *R. davurica* Pall. extracts were assessed using the DPPH method [48]. In brief, DPPH solution was freshly prepared by dissolving it in DMSO. The reaction samples were prepared by mixing 900 μL of DPPH and 100 μL of the testing specimens, distilled water (control), or DMSO alone (background). L-ascorbic acid (a most commonly used antioxidant as a standard molecule) was used as a positive control. The reaction mixtures were then incubated for 1 h in a dark place. The absorbances were determined spectrophotometrically at 517 nm (BioTek Instruments, Inc., Winooski, VT, USA). Percentage sample activity was analyzed by the following equation:

DPPH radical scavenging activity (%) = [1(A_1_ − (A_2_ − A_3_))/A_1_] × 100

where A_1_ is the absorbance of the control; A_2_ is the absorbance of the sample; and A_3_ is the absorbance of the sample background.

### 4.5. Reducing Power Assay

The reducing power capacities of *R. davurica* Pall. extracts were evaluated according to the method described in [47] with a slight modification. A solution of the sample (3.12–100 μg/mL) was prepared, and it was mixed with 2.5 mL of sodium phosphate buffer (0.2 M, pH 6.6) and 2.5 mL of potassium ferric cyanide (1%), then incubated at 50 °C for 20 min. Then, 2.5 mL of trichloroacetic acid (TCA, 10%) was added, and the mixture was centrifuged at 3000 rpm for 10 min. The upper layer (5 mL) was mixed with distilled water (5 mL) and ferric chloride (0.1%, 1 mL), and then the absorbance was measured at 700 nm using a GENios^®^ spectrophotometer (BioTek Instruments, Inc., Winooski, VT, USA). Increased absorbance indicates higher reducing power.

### 4.6. Cell Culture and Cytotoxicity Assay

C2C12 mouse skeletal muscle cells were cultured in DMEM comprising 10% heat-inactivated FBS and 100 µg/mL penicillin–streptomycin–amphotericin B solution at 37 °C in an atmosphere containing 5% CO_2_–95% air. For differentiation into myotubes, the C2C12 cell culture medium was replaced with DMEM containing 1% (*v*/*v*) horse serum for 7 days, and then the cells that had differentiated into myotubes were used for the experiments. Cytotoxicity was assessed by using the MTT assay. The cells were seeded in 24-well plates and cultured for 24 h. The *R. davurica* Pall. concentrations ranging from 0 to 1000 µg/mL were evaluated for the cytotoxicity assay by treating cells with the samples for the next 24 h. A light microscope (Olympus BX-51; Tokyo, Japan) was used to analyze the morphological changes induced by RDR treatment. Untreated cells were used as the control. MTT dye (50 µL, 5 mg/mL) was added to each well and incubated for an additional 3 h at 37 °C. After removing the supernatant, the formazan was dissolved in DMSO, and the absorbance was determined at 540 nm using a GENios^®^ spectrophotometer (BioTek Instruments, Inc., Winooski, VT, USA).

### 4.7. α-Glucosidase Inhibitory Activities

The inhibitory effect of samples on α-glucosidase activity has been measured as described elsewhere [26]. In brief, a solution containing an enzyme was prepared. Specifically, yeast α-glucosidase with an activity of 0.7 U was dissolved in a reaction buffer (pH 7.0; 100 mM of phosphate buffer (pH 7.0) containing 0.02% NaN_3_ and 0.2% BSA). As a substrate, *p*-nitrophenyl-α-D-glucopyranoside (NPG, 5 mM) was prepared in the reaction buffer. The enzyme solution (50 μL) and RDR (10 μL) of the test materials were mixed, and the absorbance was measured at 405 nm with a GENios^®^ spectrophotometer (BioTek Instruments, Inc., Winooski, VT, USA). After the addition of the substrate (50 μL), the reaction was monitored for 5 min. The increase in absorbance from zero time was measured to assess the inhibitory activities of the RDR samples. Acarbose (a substance commonly used as a standard α-glucosidase inhibitor) was used as a positive control.

α-glucosidase inhibition (%) = [1 − (C_Abs_ − S_Abs_)/(C_Abs_ − B_Abs_)] × 100

where C_Abs_ is the absorbance of the control; S_Abs_ is the absorbance of the sample; and B_Abs_ is the absorbance of the sample with no additive.

### 4.8. Measurement of Glucose Uptake

Cellular glucose uptake was assessed by the method previously described in [49,50] with a minor modification. In brief, the assay standard curve was determined using a range of glucose concentrations: 0, 10, 20, 30, 40, and 50 μg/mL, which were prepared from a working stock solution of glucose (5 mg/mL) by dilution with distilled water. For the experiment, C2C12 mouse skeletal muscle cells were gently rinsed twice with warm PBS (37 °C) and then starved in serum-free DMEM for 3 h, followed by the treatments as indicated in each figure.

The detailed procedure of the glucose uptake experiment in this study is as follows:

(1) 10 μL of the samples (or standard) was mixed with 10 μL of 4% phenol acid in an e-tube.
(2) After 5 min, 100 μL of sulfuric acid was added to the e-tube with the mixture, and then the e-tube was placed in a 30 °C water bath for 30 min.
(3) The reaction was adapted to a 96-well format, where 50 μL of the mixture was blended with 50 μL of distilled water, and the absorbance was assessed at 490 nm using a GENios^®^ spectrophotometer (BioTek Instruments, Inc., Winooski, VT, USA). The glucose uptake was quantified based on the standard curve. The results were presented as a fold-increase from their respective controls (vehicle alone).

### 4.9. Isolation of Cytosolic and Plasma Membrane Fraction Proteins

The fractions for experimental specimens were prepared as described elsewhere [51]. Briefly, the cells were lysed with buffer A [50 mM Tris, pH 8.0, 0.5 mM dithiothreitol, 0.1% (*v*/*v*) NP-40, protease inhibitors (1 mM phenylmethylsulphonyl fluoride (PMSF), 5 μg/mL leupeptin, and 5 μg/mL aprotinin), and phosphatase inhibitors (10 mM NaF and 1 mM Na_3_VO_4_)]. The cell lysate was then centrifuged at 1000× *g* for 10 min at 4 °C. The pellet was re-suspended in buffer A in ice for 10 min with intermittent vortexing, then recentrifuged at 1000× *g* for 10 min at 4 °C. The pellet was re-suspended in buffer A and placed in ice for 60 min with vortexing, then centrifuged at 16,000× *g* for 20 min at 4 °C. The supernatant was collected as the plasma membrane fraction and stored at −70 °C until use. The supernatants from the first and second spins were collected and combined, then centrifuged at 16,000× *g* for 20 min at 4 °C. The supernatant was collected, and it was then sampled as the cytosol fraction. The protein amounts in the cytosolic and membrane fractions were determined using a protein assay kit (Bio-Rad, Hercules, CA, USA).

### 4.10. Western Blotting

Cells were lysed with RIPA buffer (TransLab, Daejeon, Republic of Korea) containing a protease inhibitor cocktail in ice. The supernatant was obtained, and the protein concentration was determined using a protein assay kit (Bio-Rad, Hercules, CA, USA). The protein samples were mixed with SDS–PAGE sample buffer (62.5 mM Tris–HCl pH 6.8, 10% glycerol, 2% SDS, 0.01% bromophenol blue) and boiled for 10 min. The protein samples were separated on 12% SDS–polyacrylamide gel, then transferred to polyvinylidene fluoride (PVDF) membranes (Bio-Rad, Hercules, CA, USA). The membrane was blocked in 5% BSA. The blocked membrane was probed with specific primary antibodies and horseradish peroxidase-conjugated secondary antibodies. Chemiluminescence was detected using ChemiDoc XRS (Bio-Rad, Hercules, CA, USA). The protein bands were analyzed using the Image Lab™ software (version 5.2, Bio-Rad).

### 4.11. Animals 

Six-week-old male Sprague–Dawley rats (180–200 g body weight) were procured from Samtako BioKorea (Osan, Republic of Korea). They were acclimatized to laboratory conditions for at least 1 week before the experiment. All animals were kept at a temperature of 23.5 ± 0.5 °C and a humidity of 35–60% and were provided with water/food on a normal 12 h light–dark cycle. All experimental procedures were handled according to the guidelines of Gyeongsang National University Guide for the Care and Use of Laboratory Animals and approved by the Institutional Animal Care and Use Committee of Gyeongsang National University, with certification number GNU-161004-R0053.

### 4.12. Induction of Diabetes Rat Model and Treatment of RDR

For the induction of DM, experimental rats were fasted overnight and induced by intraperitoneal injection (IP) with streptozotocin (STZ; 45 mg/kg), which was freshly prepared in citrate buffer (0.1 M, pH 4.5). The status of diabetic rats was examined by the analysis of plasma glucose levels, determined at 72 h and again on day 7 after the STZ injection. A Roche Accu-Chek glucometer (Roche Diagnostics, Mannheim, Germany) was used to determine the fasting blood glucose. Blood glucose levels higher than 250 mg/dL were used to identify diabetic rats in the present study. The rats were randomly assigned into five groups (*n* = 5 for each group). These groups were divided as follows:

Group I—Control (normal rats administered with saline)
Group II—STZ (diabetic rats administered with saline)
Group III—STZ + RDR (diabetic rats administered with a low dosage of RDR, 100 mg/kg)
Group IV—STZ + RDR (diabetic rats administered with a high dosage of RDR, 500 mg/kg)
Group V—STZ + MET (diabetic rats administered with metformin, 200 mg/kg).

RDR and MET were administered orally using an intragastric tube during the experiments. During the experiment, body weight changes, food intake, and water consumption were recorded daily and/or weekly.

### 4.13. Blood Biochemical Analysis

For the assay of blood biochemicals, the rats were fasted for 12 h, and then the blood was collected. The heparin-processed tubes were immediately used to collect blood samples. Plasma samples were collected by the centrifugation of whole blood at 1600× *g* for 15 min at 4 °C, and this separation procedure was performed within 30 min. Aliquots of plasma samples were collected for insulin, adiponectin, and leptin assays. The plasma levels of glucose, ALT, AST, BUN, and creatinine were determined colorimetrically using commercial diagnostic kits with the IDEXX VetTest Chemistry Analyzer (IDEXX laboratories, Inc., Westbrook, ME, USA). Plasma insulin was assayed at the end of experiments by using a rat insulin ELISA kit (SHIBAYAGI, Co., Ltd., Gunma, Japan) with a spectrophotometric microplate reader (BioTek Instruments, Inc., Winooski, VT, USA).

### 4.14. Histological Analysis

Before sacrifice, pancreas tissues from all groups were subsequently fixed in 10% neutralized formalin solution. After 24 h, the tissues were dehydrated in escalating ethanol, cleaned in xylene, and finally embedded in paraffin wax. The paraffin blocks were serially sectioned into 5 μm thickness and then stained with hematoxylin and eosin (H&E) to observe any histological changes. Representative sections were evaluated for histopathological alterations using a light microscope (Olympus BX-51; Tokyo, Japan).

### 4.15. GC Analysis

The chemical constituents in RDR were identified by the method previously described [52]. RDR was analyzed using the mass spectrometry (MS) fragmentation pattern of each component and the Kovats gas chromatographic retention index (KI) (KOVATS, 1965). A 30 m × 0.25 mm i.d. (*d*_f_ = 0.25 μm) DB-WAX bounded-phase fused-silica capillary column (Agilent, Folsom, CA, USA) was utilized for the analysis of RDR. This column was interfaced with an Agilent 5971A mass selective detector (GC/MS), which allowed for the mass spectral identification of the GC components at an MS ionization voltage of 70 eV. In splitless mode, the helium carrier gas’s linear velocity was 30 cm/s. The temperatures of the injector and detector were set at 250 °C. The oven was programmed to increase its temperature at a rate of 4 °C per minute between 60 and 140 °C. GC column conditions were exactly the same as those used for GC/FID. Interpretation of mass spectrum from GC–MS was performed using the database of the National Institute of Standards and Technology (NIST). The names and structures of the components of RDR were ascertained.

### 4.16. Molecular Docking Analysis

The chemical structures of the eight compounds in RDR were obtained from the PubChem database (https://pubchem.ncbi.nlm.nih.gov) to evaluate the results of the molecular docking study. After that, the AutoDock Vina program version 1.1.2 was used to screen the binding site of the co-crystallized structure of the protein GLUT4 (PDB ID: 5IFS) for structure-based screening. Because AutoDock Vina performs better than AutoDock 4.0, it was selected as the recommended docking program. It has advantages including quicker processing speed, automated pre-calculation of grid maps, and improved mean precision in binding mode prediction, all of which are performed internally [53]. The protein was converted from PDB to PDBQT format using Open Babel version 2.3, and Kollman charges were applied to incorporate polar hydrogen atoms and charges. Grid parameters were set with XYZ dimensions of 40 Å × 25 Å × 40 Å and a spacing of 0.375 Å around the protein. Each ligand was docked to generate ten poses per compound, which were then clustered to identify the optimal binding pose based on binding energy and RMSD. A Python script from the Vina manual (https://vina.scripps.edu/manual/) was used to select compounds with high dock scores indicating favorable binding energies. The chemical structures of the eight compounds from the PubChem database (https://pubchem.ncbi.nlm.nih.gov/) were docked using AutoDock Vina in PDB format. The PyMOL (version 2.4.0) and Discovery Studio software (https://discover.3ds.com/discovery-studio-visualizer-download, accessed on 26 June 2024) were used to visualize and analyze the data once the docking trials were completed.

### 4.17. Statistical Analysis

The data are presented as mean ± standard deviation (SD). A paired Student’s *t*-test was used to assess the significance of differences between the two mean values. Statistical significance was set at * *p* < 0.05 and ** *p* < 0.01.

## 5. Conclusions

In summary, our study highlights the potent antioxidant activity and glucose homeostasis regulatory effects of RDR (the root extract of *R. davurica* Pall.). This natural extract plays a crucial role in inhibiting α-glucosidase activity, contributing to its antidiabetic properties. Furthermore, RDR significantly promotes glucose uptake by facilitating GLUT4 translocation, a process mediated through the activation of the AMPK signaling pathways in C2C12 skeletal muscle cells. Notably, molecular docking studies of glucose transporter GLUT4 with RDR components revealed a significantly high binding score, emphasizing their structural compatibility. The observed improvement in diabetic symptoms in STZ-induced diabetic rats further underscores the protective effects of RDR against diabetes. Our findings suggest that RDR treatment holds promise as an effective approach for managing diabetes. By delving into the pharmacological and bioactive properties, along with the underlying mechanisms governing RDR’s impact on glucose metabolism, our study contributes to a deeper understanding of its potential therapeutic applications, particularly in the prevention and treatment of diabetes mellitus.

## Figures and Tables

**Table 1:** Compounds and their biological activities identified in RDR.

No.	Retention Time (min)	Compound Name	Molecular Formula	Score (%)	Mass (*m*/*z*)	Activity ^(1)^
**1**	24.358	4H-Pyran-4-one, 2,3-dihydro-3,5-dihydroxy-6-methyl-(=DDMP)	C_6_H_8_O_4_	71.94	144	Antioxidant, antidiabetic, antimicrobial, anti-inflammatory
**2**	35.195	1,2,3-Benzenetriol (=Pyrogallol)	C_6_H_6_O_3_	94.58	126	Antimicrobial preservative
**3**	40.980	D-Allose	C_6_H_12_O_6_	79.29	180.1	Antioxidant, antidiabetic, anticancer,
**4**	55.805	Dibutyl phthalate	C_16_H_22_O_4_	95.45	278.2	Antifouling, antimicrobial
**5**	56.027	n-Hexadecanoic acid (=Palmitic acid)	C_16_H_32_O_2_	92.52	256.2	Antioxidant, hypocholesterolemic nematicide, pesticide, lubricant, antiandrogenic, flavor, hemolytic 5-alpha reductase inhibitor
**6**	59.157	9,12-Octadecadienoic acid (Z,Z)- (=Linoleic acid)	C_18_H_32_O_2_	96.31	280.2	Antioxidant, antidiabetic, anticancer, hypocholesterolemic action
**7**	59.289	Oleic acid	C_18_H_34_O_2_	89.19	282.3	Anti-inflammatory, anti-androgenic cancer preventive, dermatitigenic hypocholesterolemic, 5-alpha reductase inhibitor, anemia genic insectifuge, flavor
**8**	59.799	Octadecanoic acid (=Stearic acid)	C_18_H_36_O_2_	89.09	284.3	Antidiabetic, hypocholesterolemic action

^(1)^ Dr. Duke’s phytochemical and ethnobotanical database.

**Table 2:** Molecular docking of GLUT4 with the main chemical compounds in RDR.

Compound Name	AutoDock Score (kcal/mol)	Hydrogen Bond Interactions	Hydrophobic Interactions
4H-Pyran-4-one, 2,3-dihydro-3,5-dihydroxy-6-methyl-(=DDMP)	−5.5	384-HIS, 386-LYS, 387-ASN, 480-GLY	479-ILE
1,2,3-Benzenetriol (=Pyrogallol)	−5.6	36-ASN, 144-ARG	-
D-Allose	−6.9	56-THR, 110-TYR, 400-ARG	-
Dibutyl phthalate	−5.7	56-THR	110-TYR, 143-TYR, 175-ILE, 327-PRO, 396-LEU, 400-ARG, 402-VAL, 483-PRO
n-Hexadecanoic acid (=Palmitic acid)	−6.3	54-GLY, 436-THR	70-ILE, 110-TYR, 175-ILE, 327-PRO, 396-LEU, 402-VAL, 459-LEU
9,12-Octadecadienoic acid (Z,Z)- (=Linoleic acid)	−6.0	56-THR	106-PRO, 110-TYR, 175-ILE, 327-PRO, 396-LEU, 402-VAL, 459-LEU, 483-PRO
Oleic acid	−5.7	-	110-TYR, 327-PRO, 396-LEU, 400-ARG, 402-VAL, 459-LEU, 483-PRO
Octadecanoic acid (=Stearic acid)	−5.7	110-TYR	175-ILE, 327-PRO, 396-LEU, 400-ARG, 402-VAL, 459-LEU, 483-PRO

## Data Availability

Data is contained within the article and Supplementary Materials.

## Supplementary Materials

The following supporting information can be downloaded at: https://www.mdpi.com/article/10.3390/ijms25168944/s1.
