# Supplementary material for: Therapeutic Potential of Rosa davurica Pall. Root Extract as an Antidiabetic Agent: A Comprehensive Analysis from Molecular Mechanisms to In Vivo Efficacy
Source: Int J Mol Sci. 2024 Aug 16;25(16):8944. doi: 10.3390/ijms25168944 (PMC11354915; doi:10.3390/ijms25168944)

1

4H-Pyran-4-one, 2,3-dihydro-3,5-dihydroxy-6-methyl-

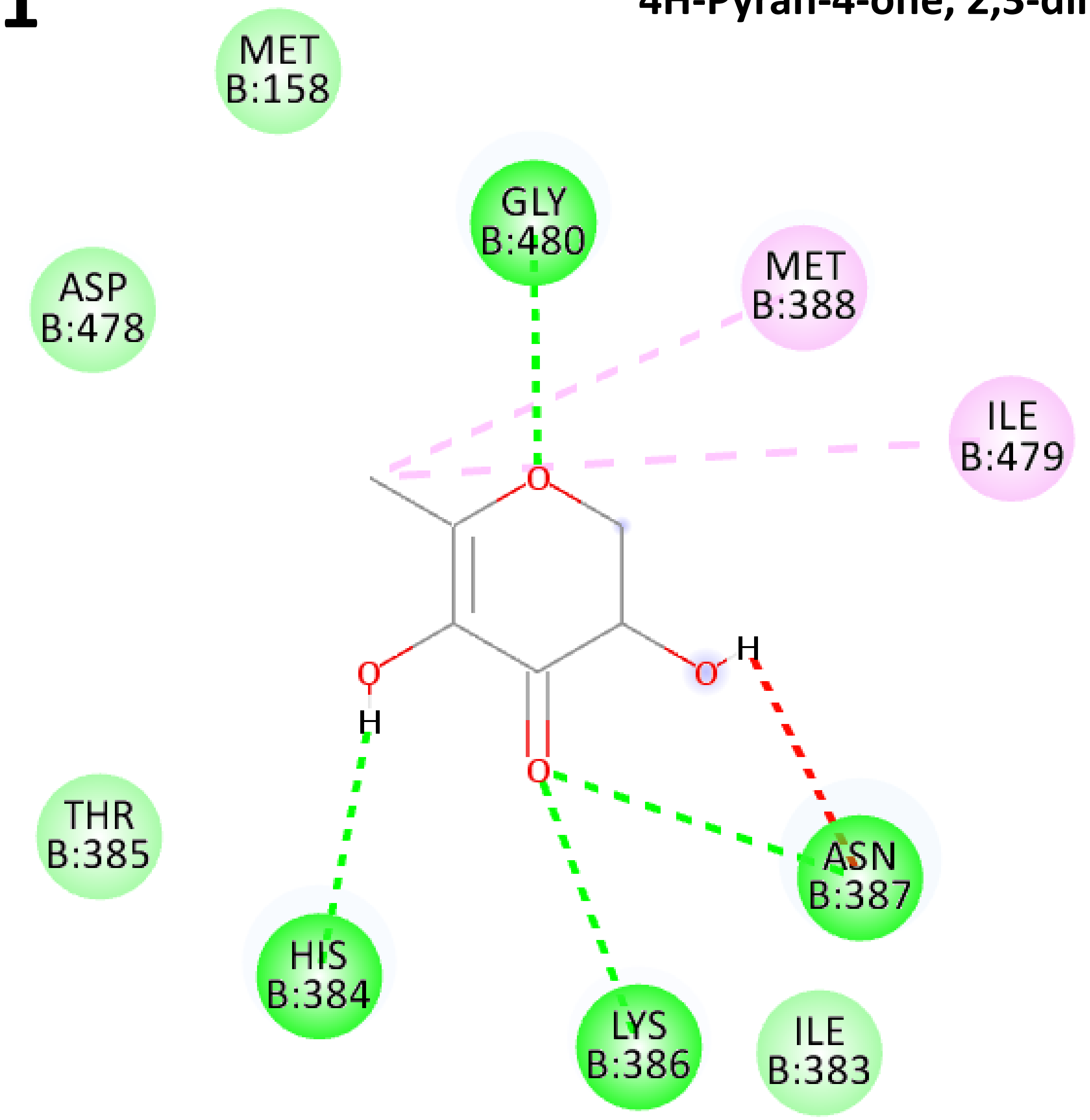

Interactions

van der Waals

Conventional Hydrogen Bond

Unfavorable Donor-Donor

Alkyl

2 Pyrogallol

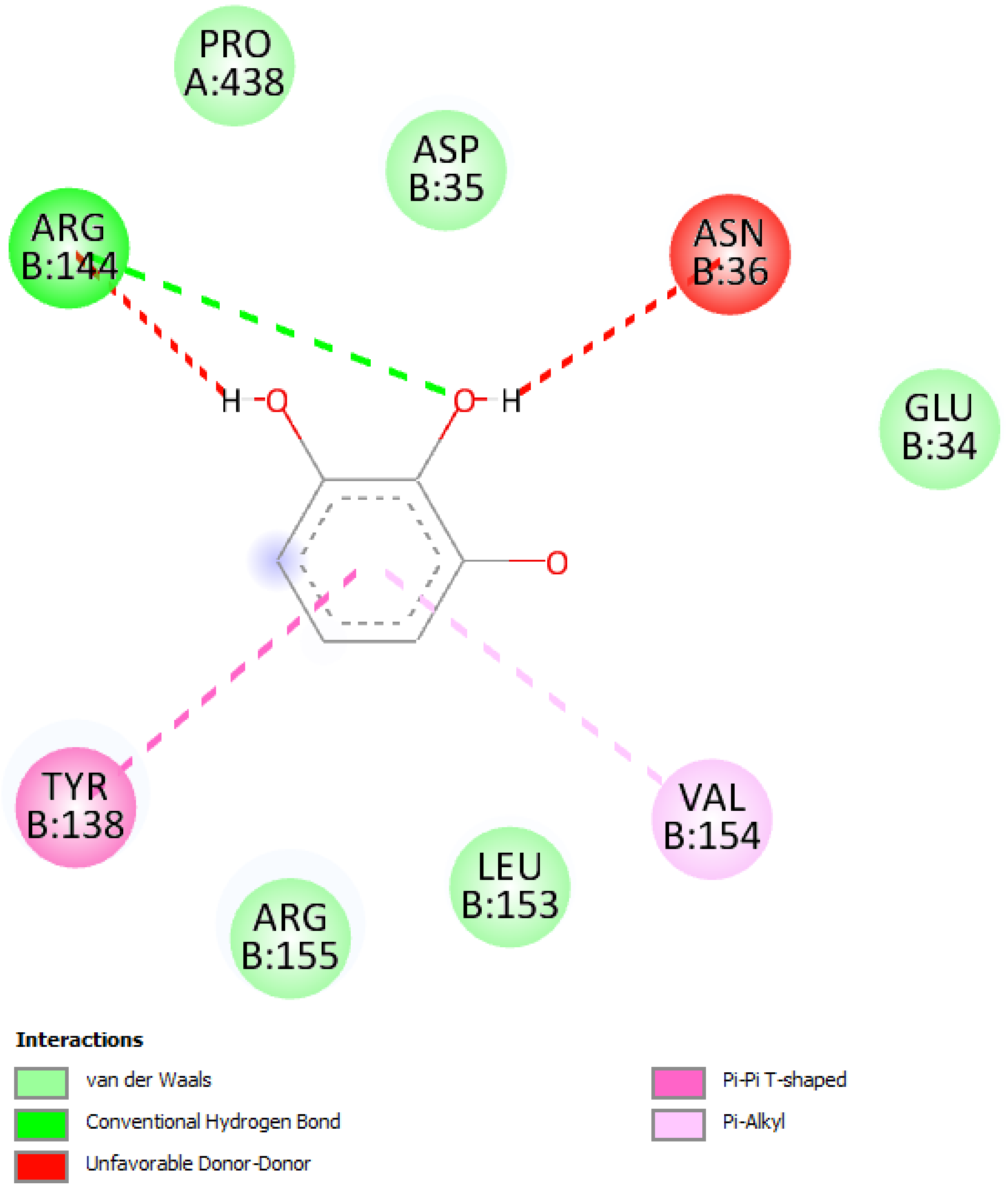

3 D-Allose

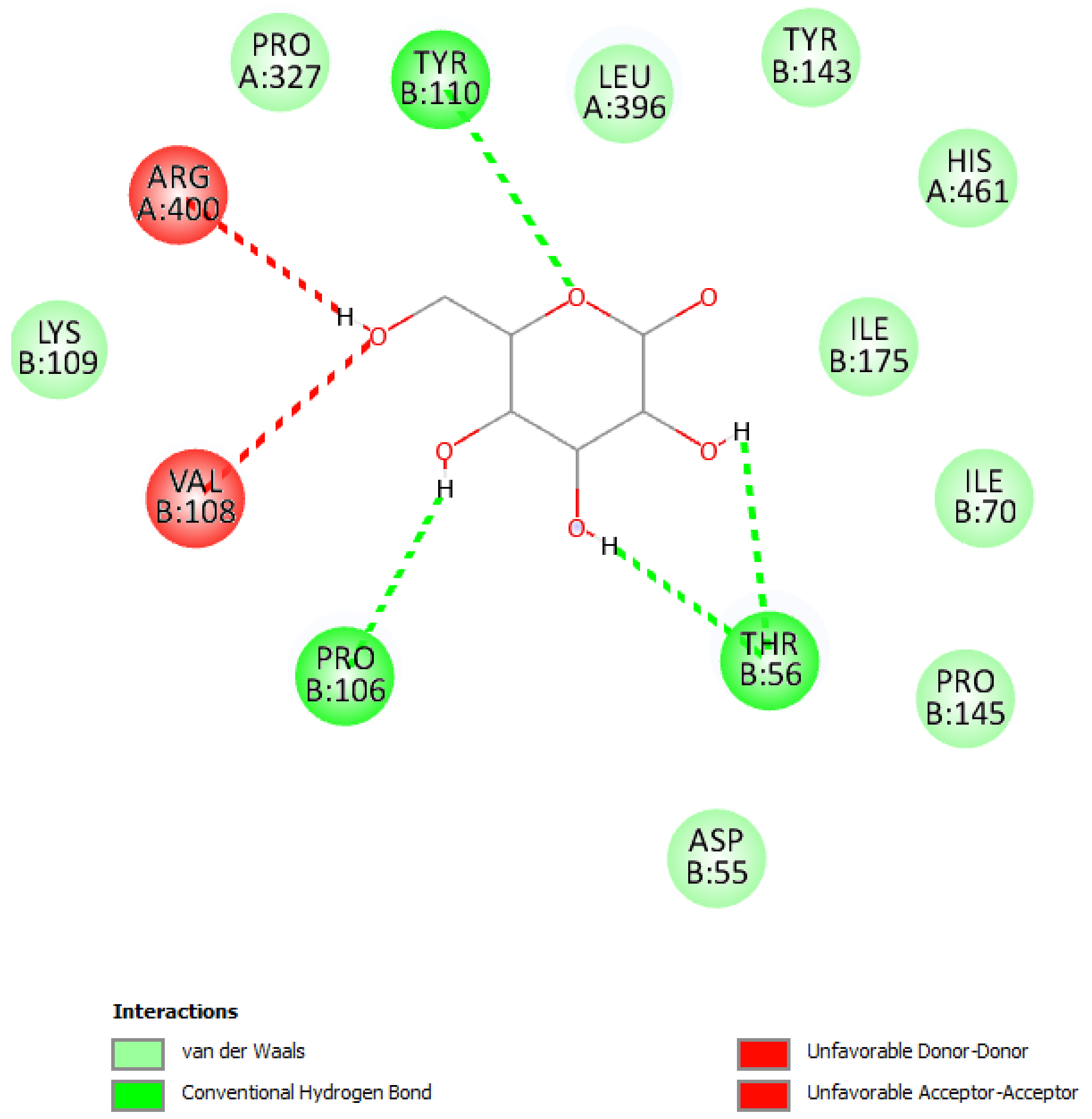
PRO A:327
TYR B:110
LEU A:396
TYR B:143
HIS A:461
ARG A:400
LYS B:109
ILE B:175
VAL B:108
ILE B:70
PRO B:106
THR B:56
PRO B:145
ASP B:55
Interactions
van der Waals
Conventional Hydrogen Bond
Unfavorable Donor-Donor
Unfavorable Acceptor-Acceptor

Dibutyl Phthalate

4

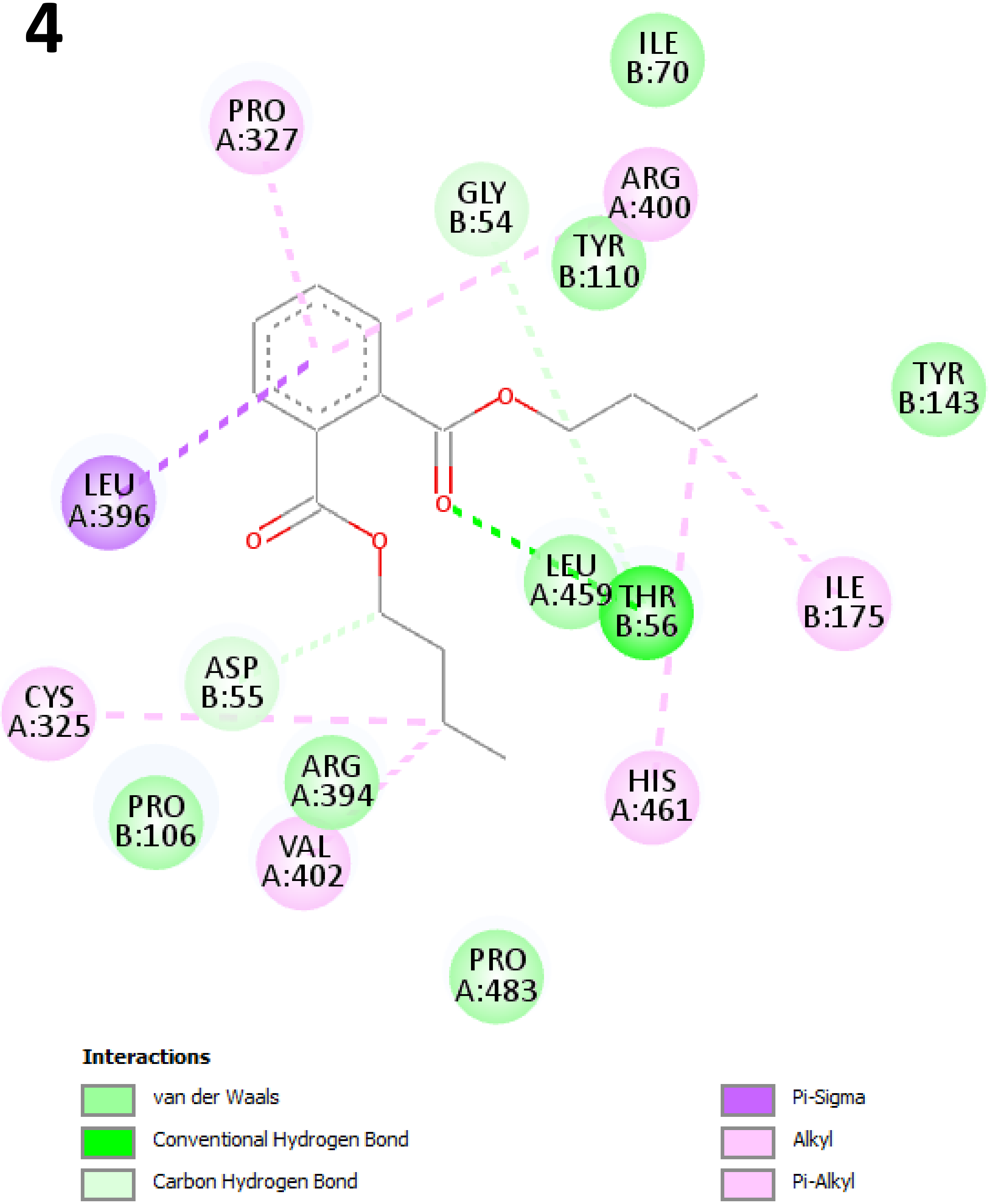

5

Palmitic Acid

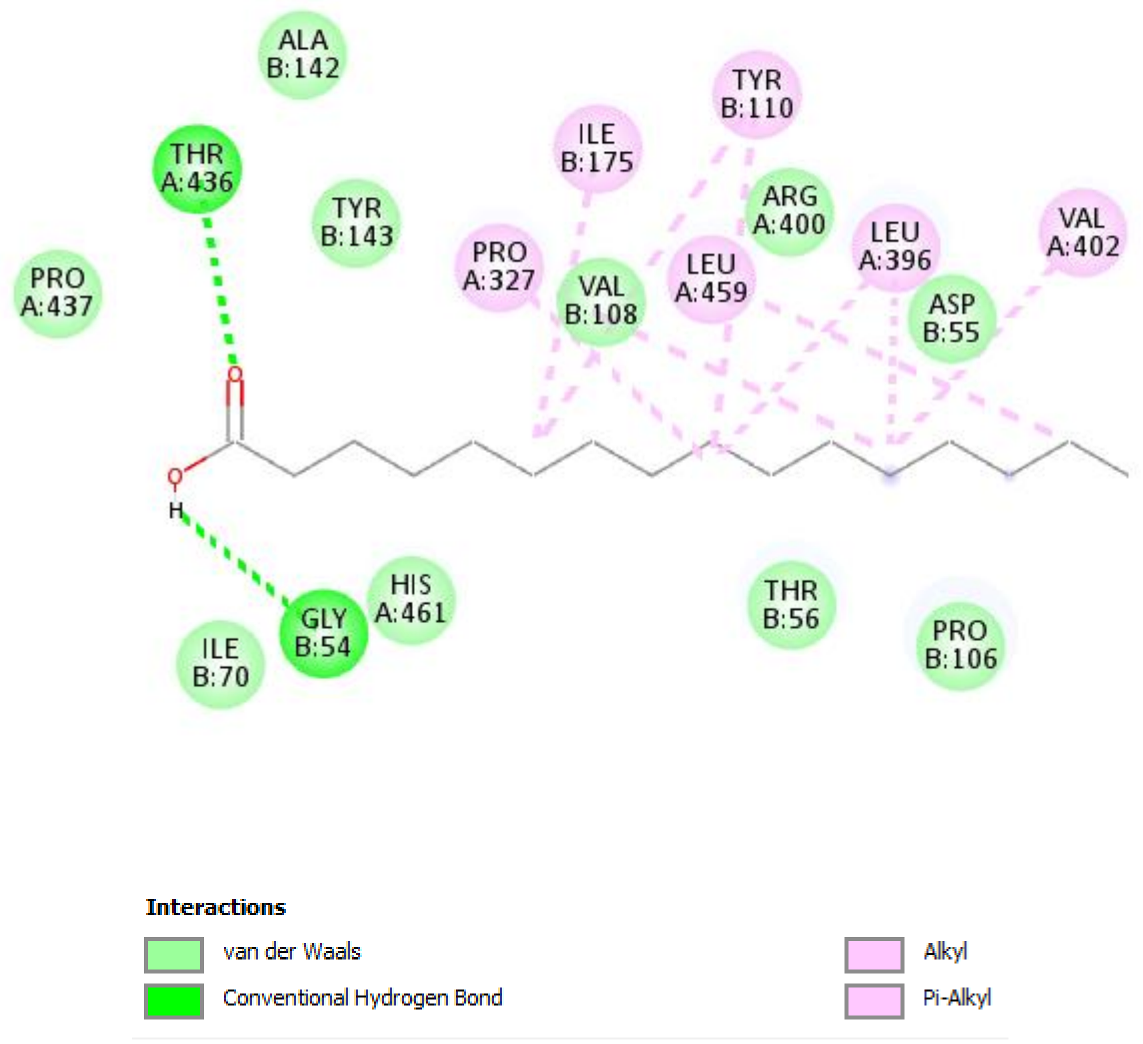

6

5280

Linoleic Acid

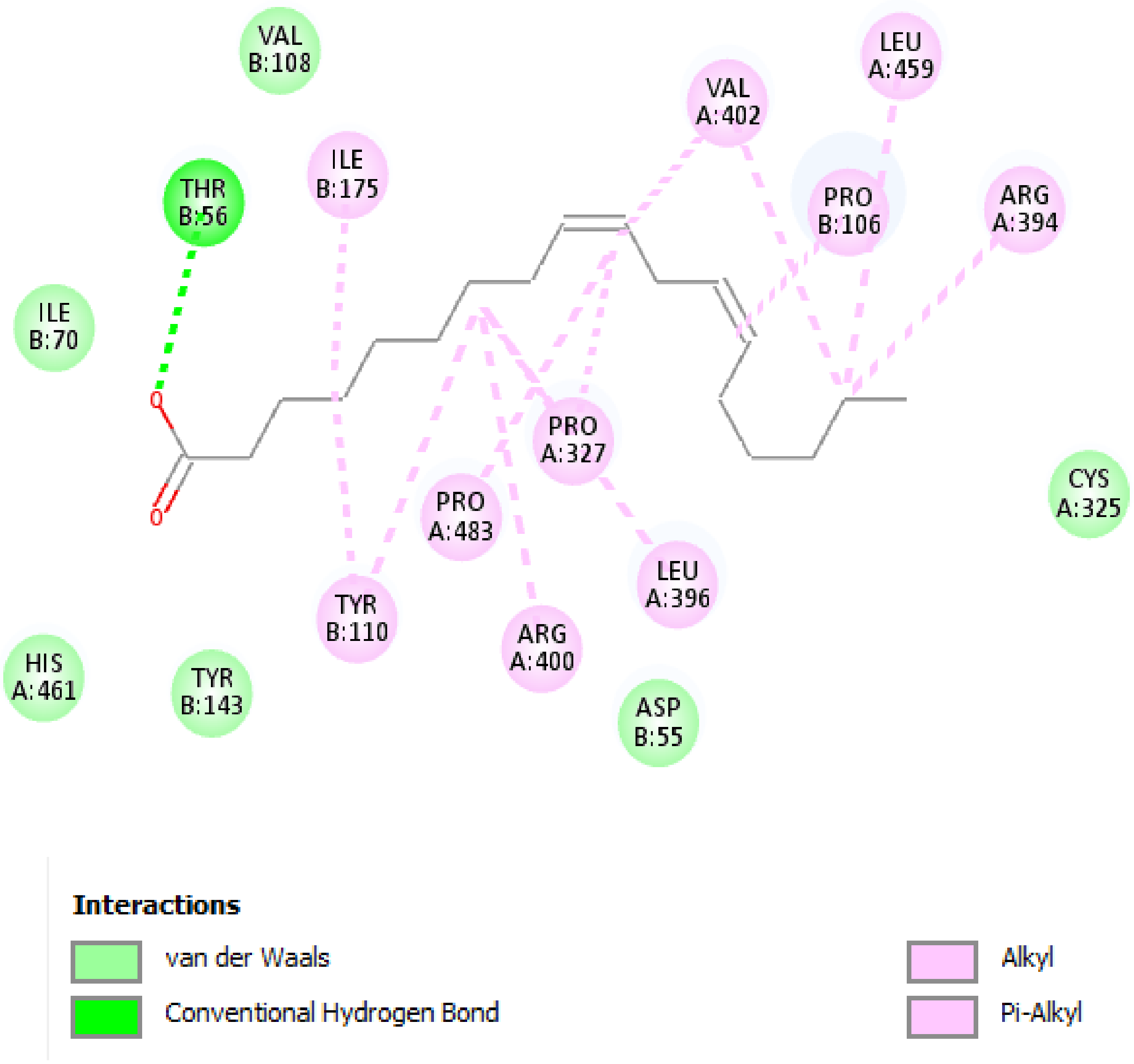

7 Oleic acid

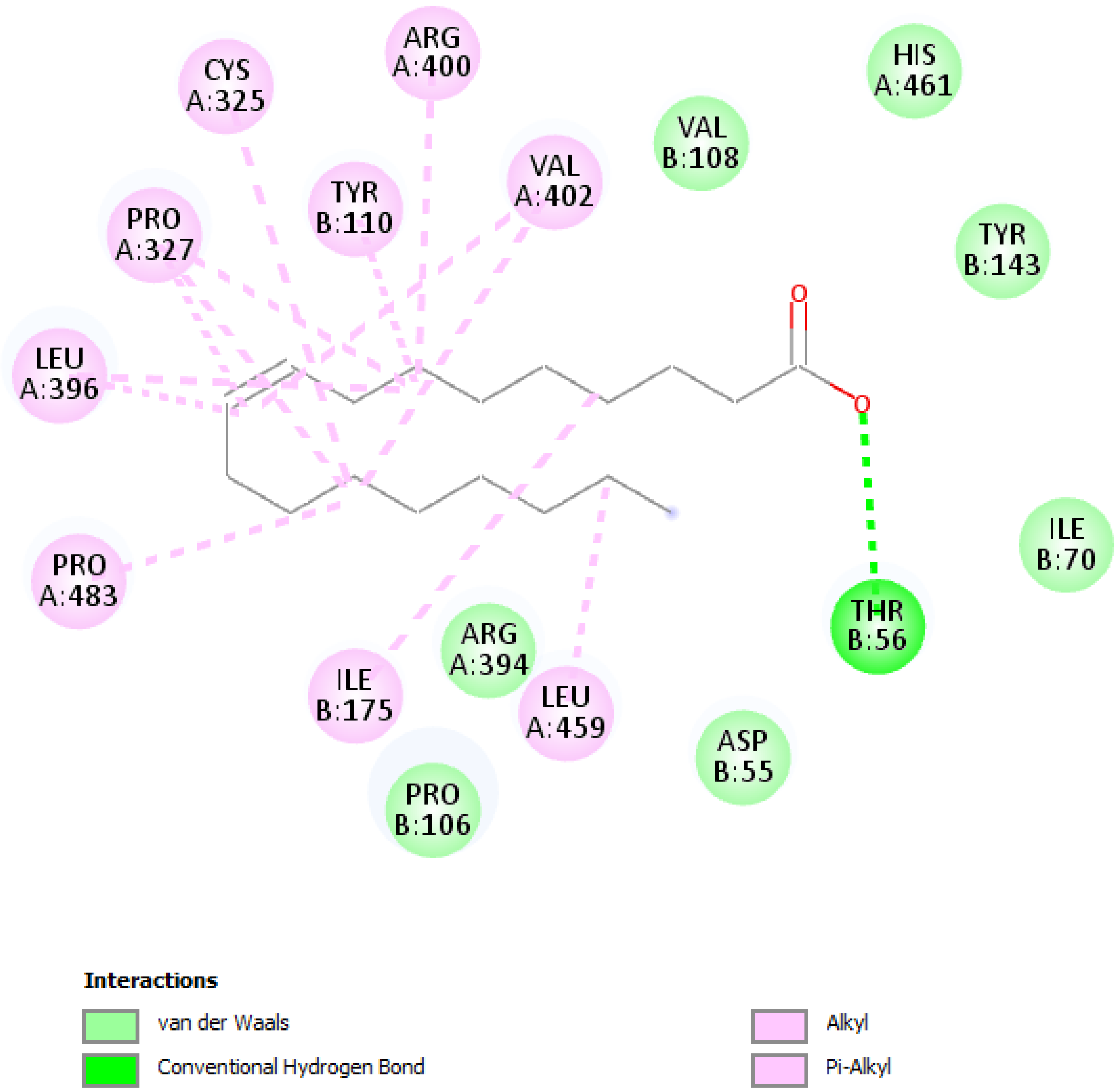
ARG A:400
CYS A:325
HIS A:461
VAL B:108
VAL A:402
TYR B:110
PRO A:327
TYR B:143
O
LEU A:396
O
ILE B:70
PRO A:483
THR B:56
ARG A:394
ILE B:175
LEU A:459
ASP B:55
PRO B:106
Interactions
van der Waals
Conventional Hydrogen Bond
Alkyl
Pi-Alkyl

8 Stearic acid

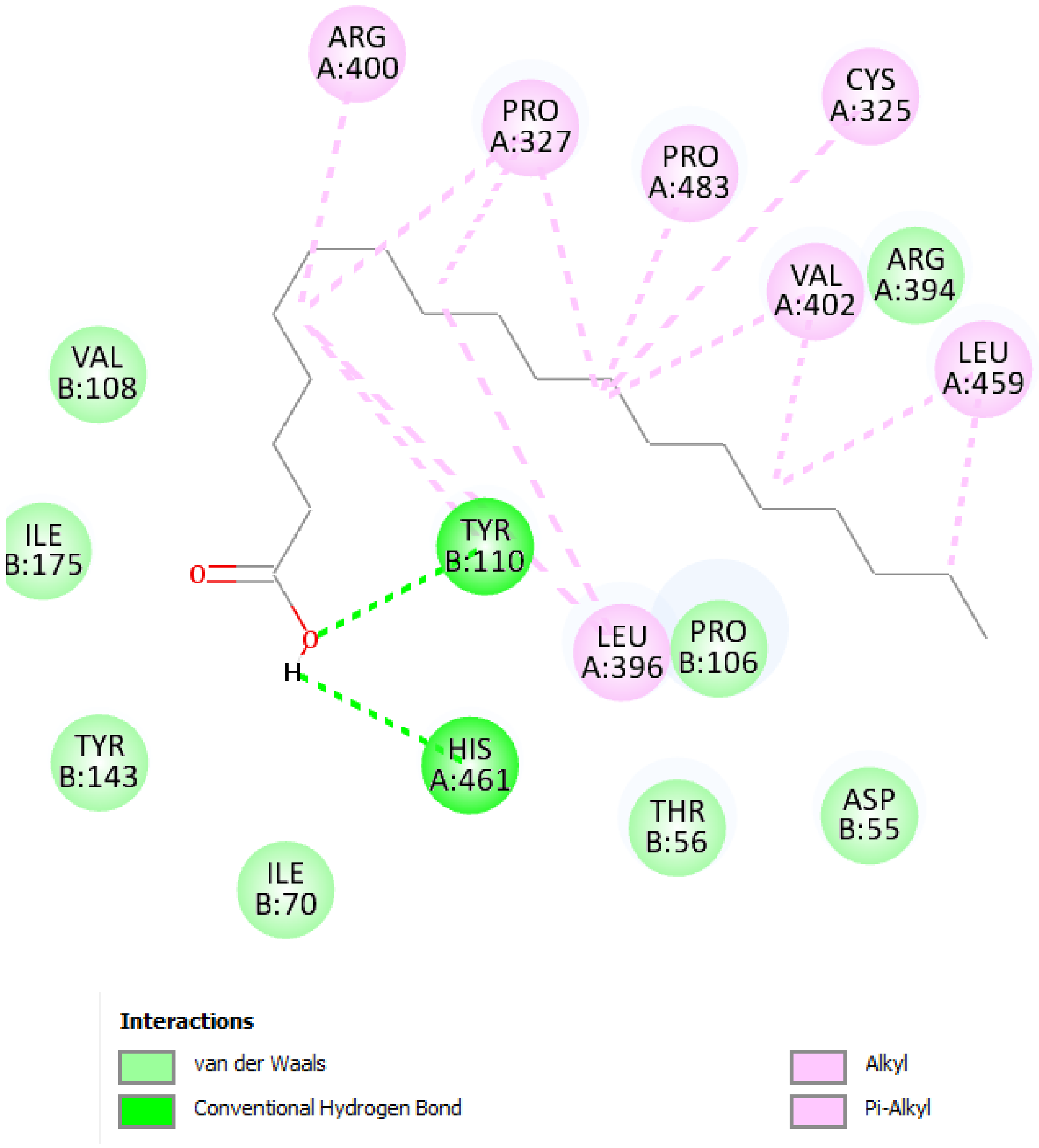

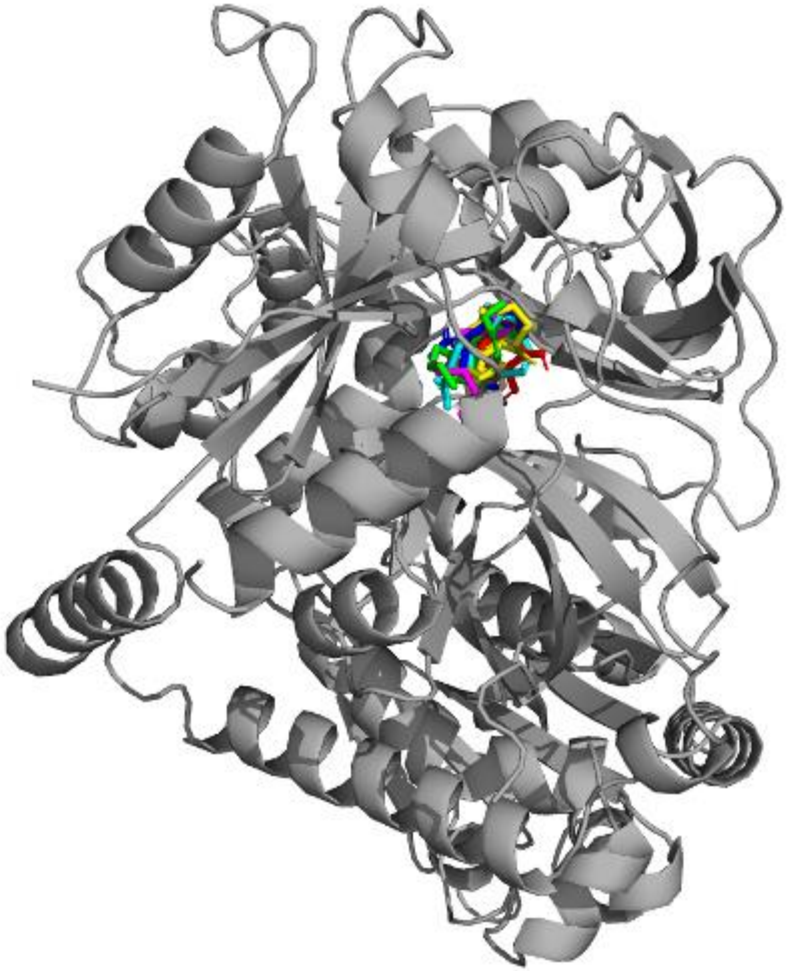

1

Tint	119838

4H-Pyran-4-one, 2,3-dihydro-3,5-dihydroxy-6-methyl-

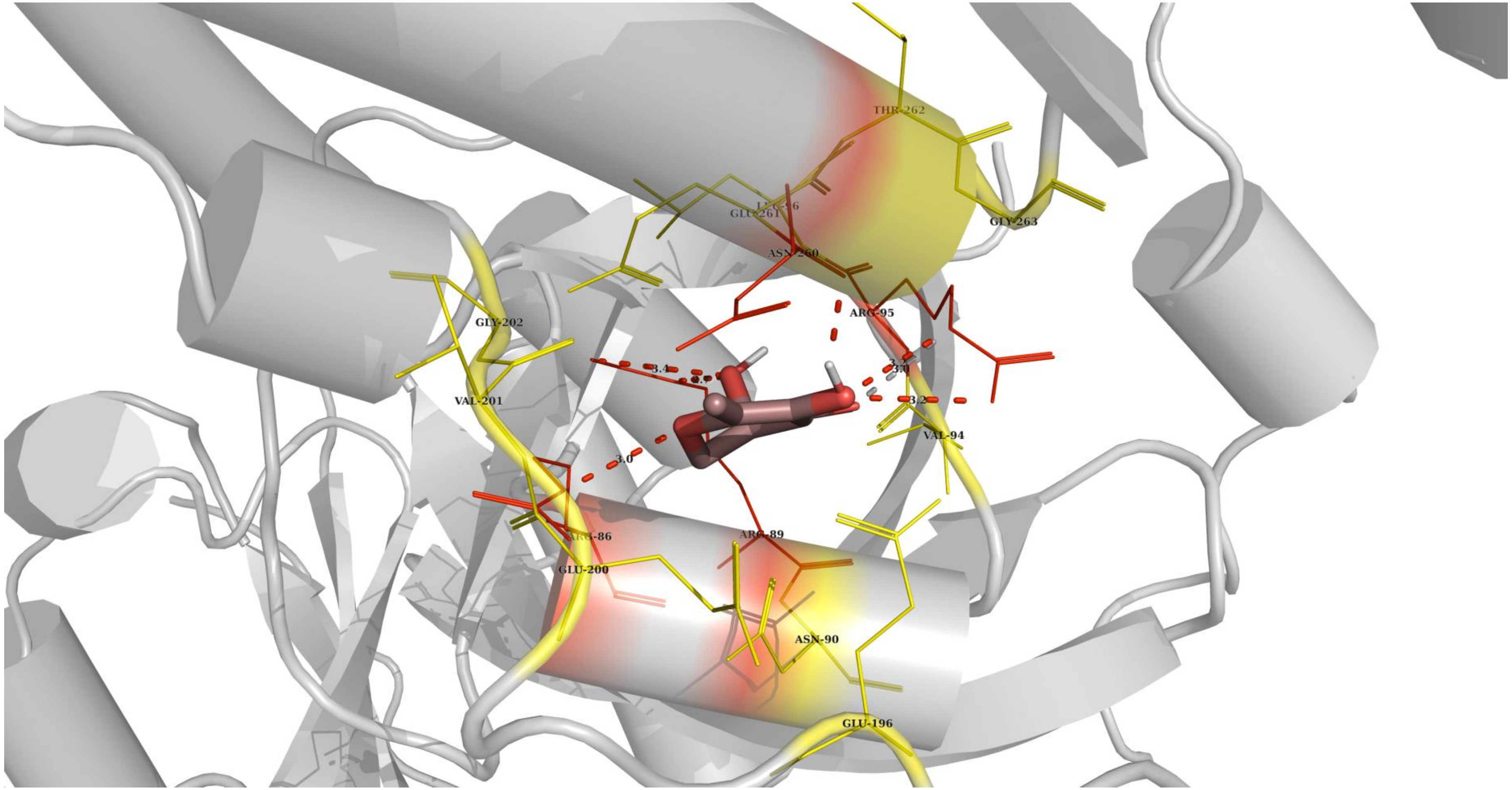

2

Orange	1057

Pyrogallol

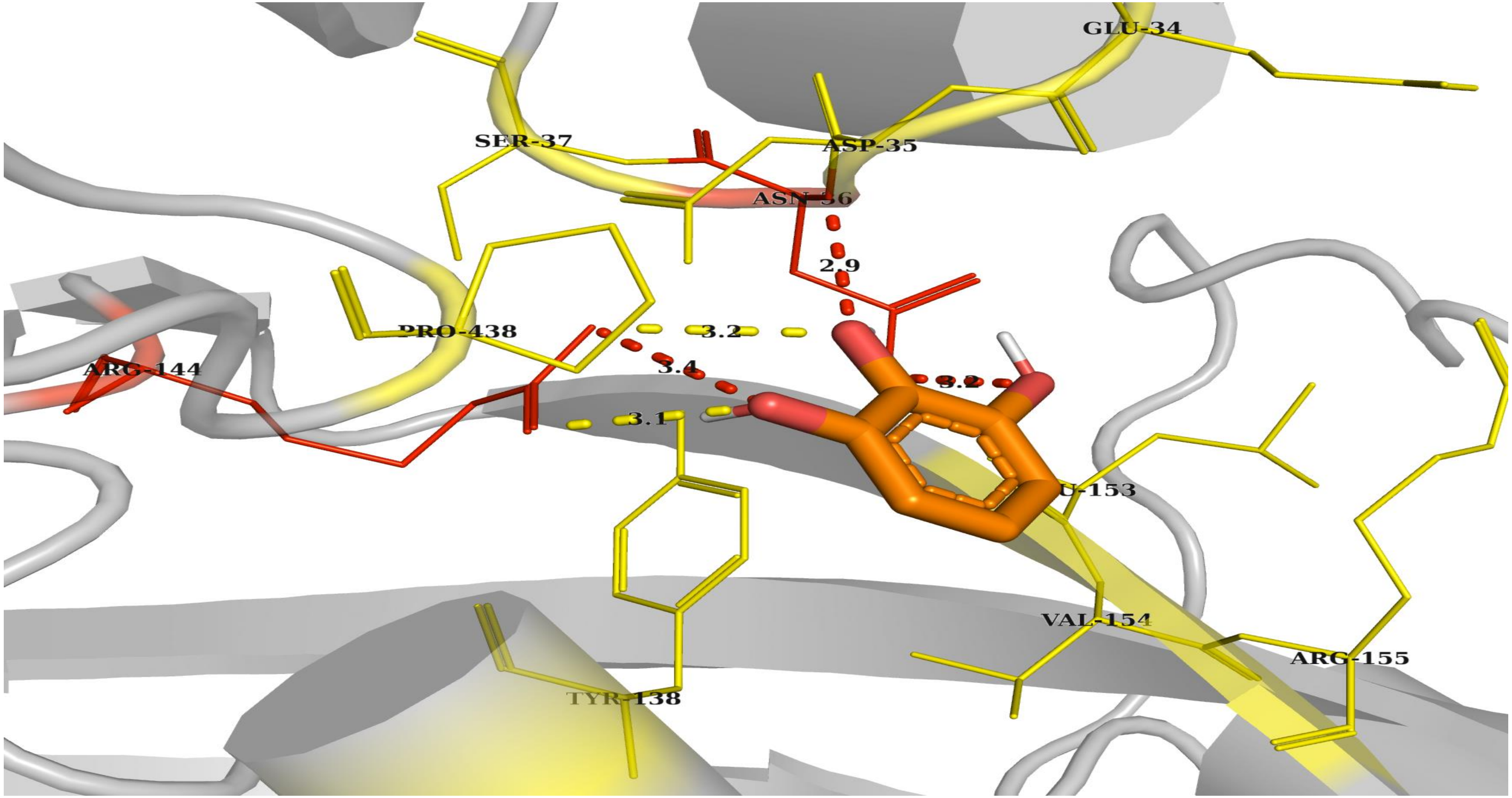

3

Red	439507

D-Allose

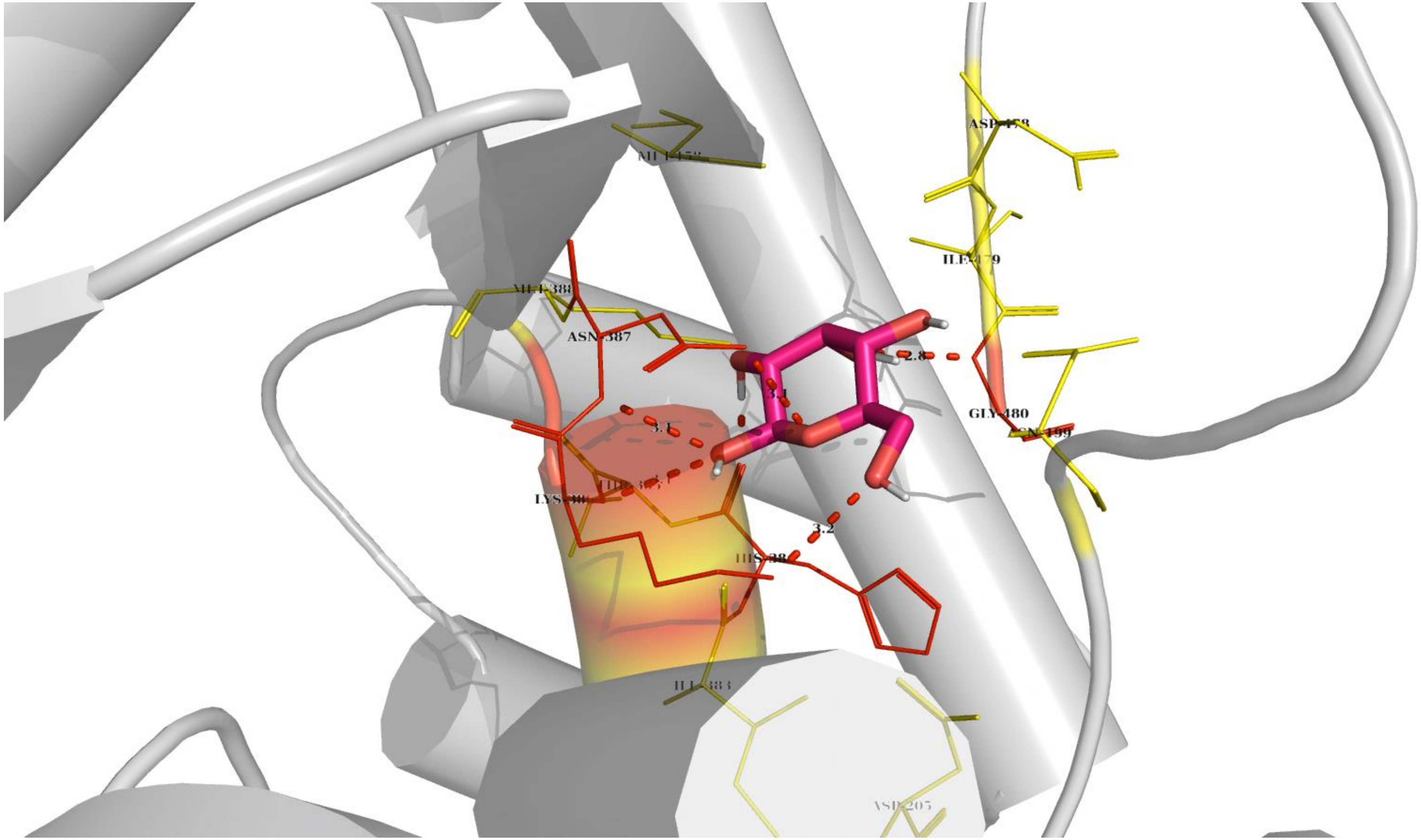

4

**Yellow**	3026

Dibutyl Phthalate

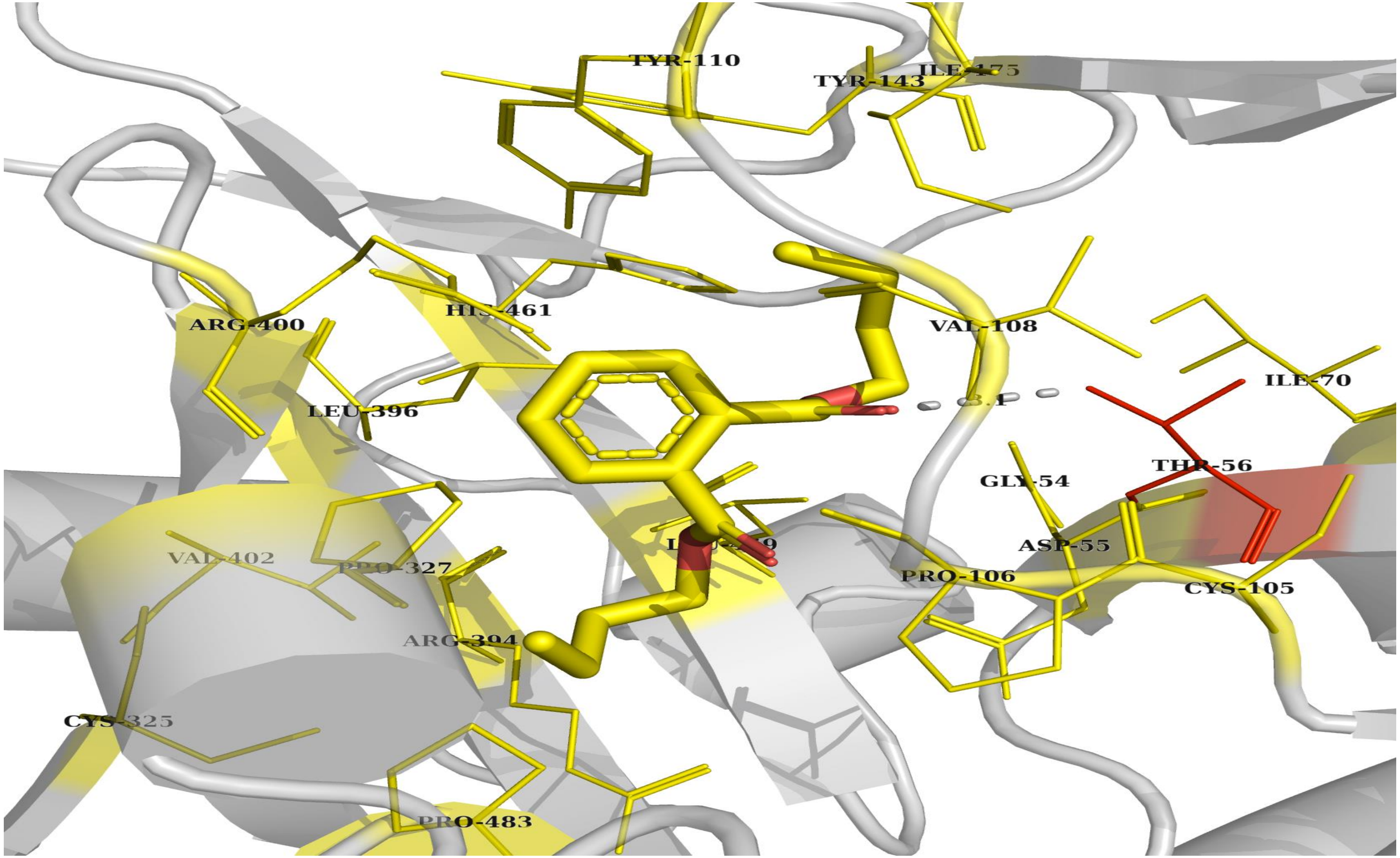

5

**Blue**	985	Palmitic Acid

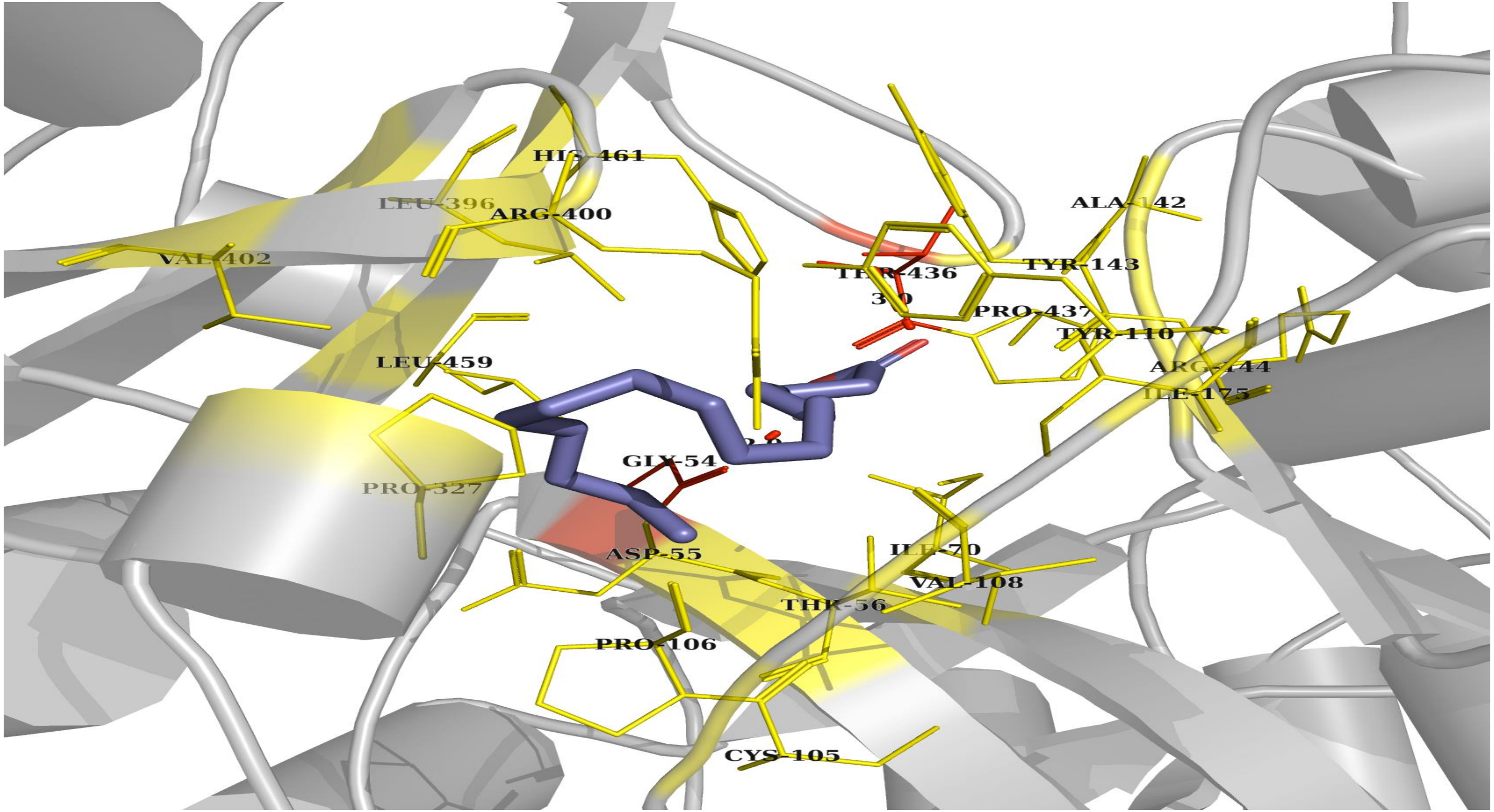

6

**Cyan**	5280450

Linoleic Acid

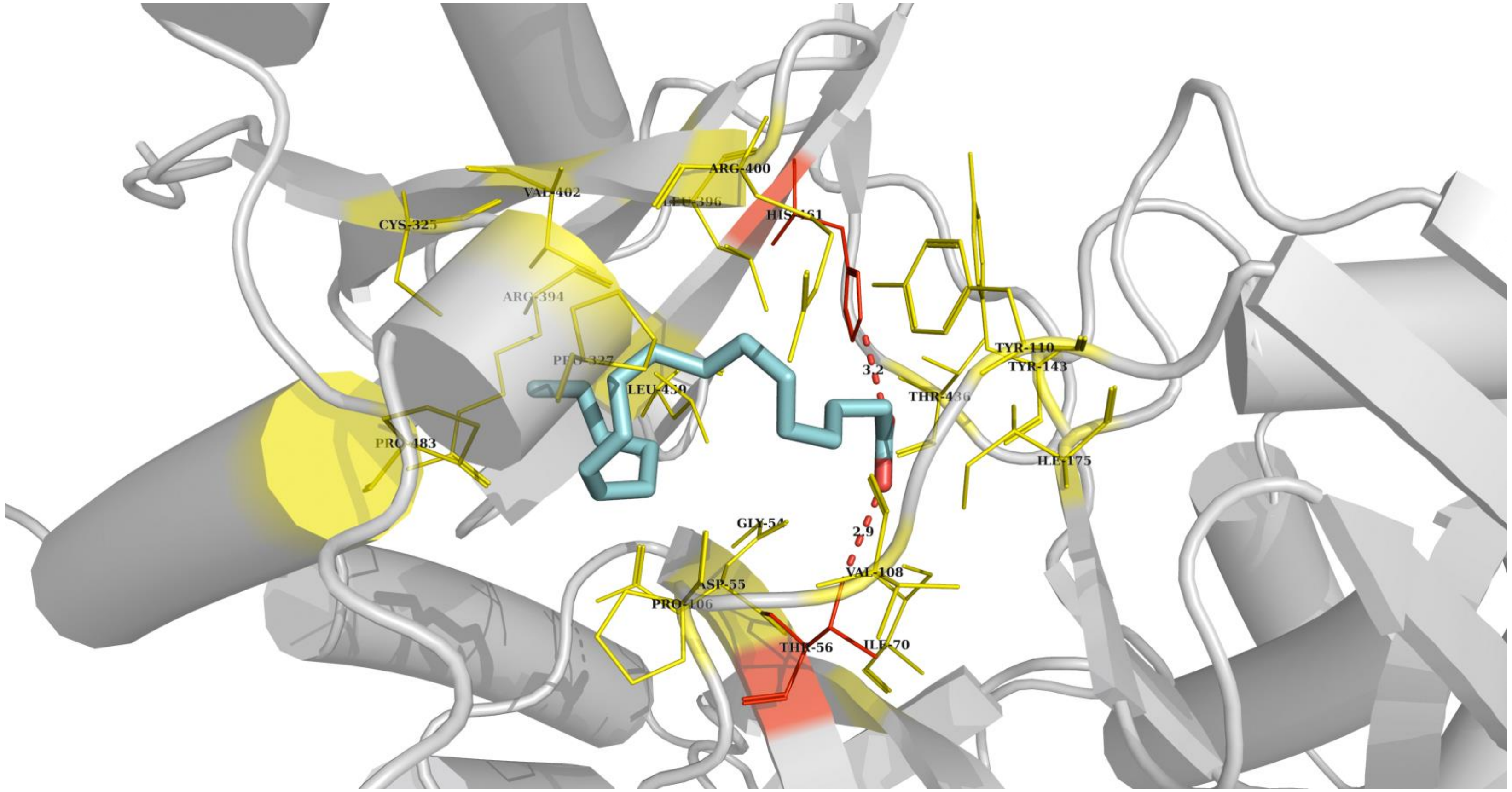

7

Green	445639

Oleic acid

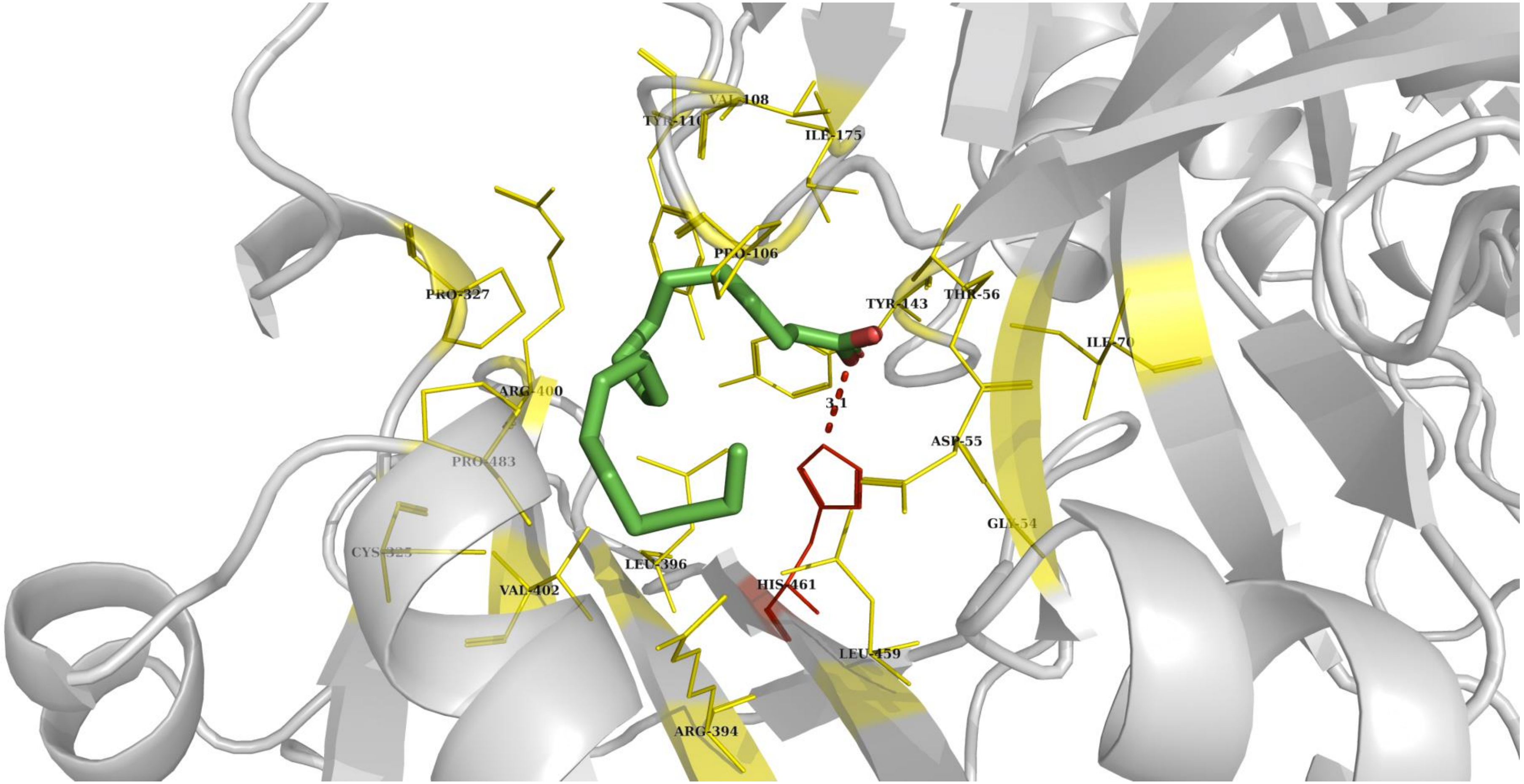

8

**Magenta**	5281	Stearic acid

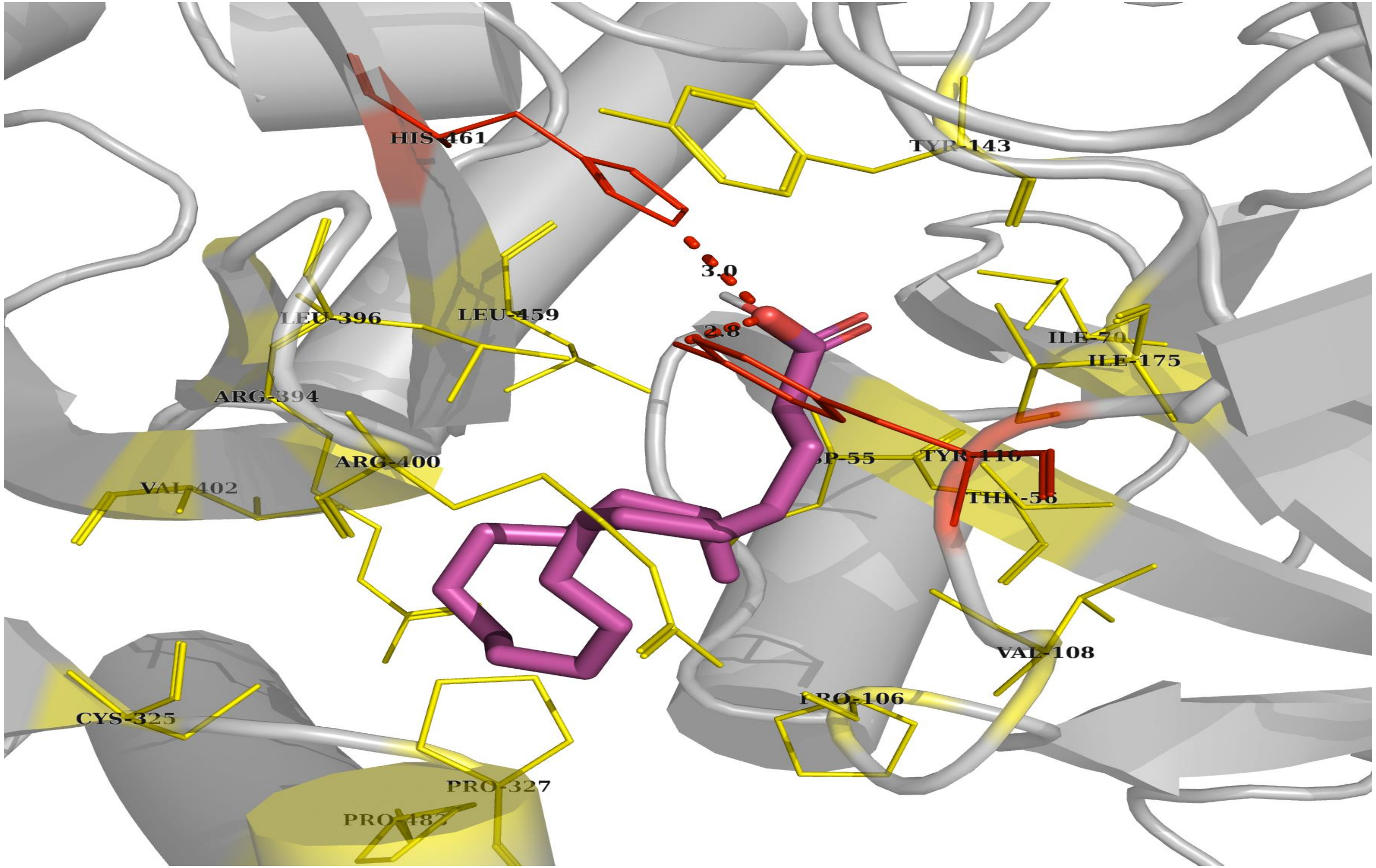

Supplement: Supplementary file 1 [file ijms-25-08944-s001.zip › Molecular docking-SFig data final.pdf]
